# Portuguese Common Bean Natural Variation Helps to Clarify the Genetic Architecture of the Legume’s Nutritional Composition and Protein Quality

**DOI:** 10.3390/plants11010026

**Published:** 2021-12-22

**Authors:** Francisco A. Mendes, Susana T. Leitão, Verónica Correia, Elsa Mecha, Diego Rubiales, Maria Rosário Bronze, Maria Carlota Vaz Patto

**Affiliations:** 1 Instituto de Tecnologia Química e Biológica António Xavier, Universidade Nova de Lisboa, Av. da República, 2780-157 Oeiras, Portugal; famendes@itqb.unl.pt (F.A.M.); vero_correi@hotmail.com (V.C.); emecha@itqb.unl.pt (E.M.); mbronze@ibet.pt (M.R.B.); cpatto@itqb.unl.pt (M.C.V.P.); 2 Faculdade de Farmácia, Universidade de Lisboa, 1649-019 Lisboa, Portugal; 3 iBET—Instituto de Biologia Experimental e Tecnológica, Av. da República, 2780-157 Oeiras, Portugal; 4 Instituto de Agricultura Sostenible, CSIC, Av. Menéndez Pidal, 14004 Cordova, Spain; diego.rubiales@ias.csic.es

**Keywords:** ash, amino acids, carbohydrates, fat, fiber, GWAS, nutritional quality, *Phaseolus vulgaris*, resistant starch, trypsin inhibitor

## Abstract

Common bean is a nutritious food legume widely appreciated by consumers worldwide. It is a staple food in Latin America, and a component of the Mediterranean diet, being an affordable source of protein with high potential as a gourmet food. Breeding for nutritional quality, including both macro and micronutrients, and meeting organoleptic consumers’ preferences is a difficult task which is facilitated by uncovering the genetic basis of related traits. This study explored the diversity of 106 Portuguese common bean accessions, under two contrasting environments, to gain insight into the genetic basis of nutritional composition (ash, carbohydrates, fat, fiber, moisture, protein, and resistant starch contents) and protein quality (amino acid contents and trypsin inhibitor activity) traits through a genome-wide association study. Single-nucleotide polymorphism-trait associations were tested using linear mixed models accounting for the accessions’ genetic relatedness. Mapping resolution to the gene level was achieved in 56% of the cases, with 102 candidate genes proposed for 136 genomic regions associated with trait variation. Only one marker-trait association was stable across environments, highlighting the associations’ environment-specific nature and the importance of genotype × environment interaction for crops’ local adaptation and quality. This study provides novel information to better understand the molecular mechanisms regulating the nutritional quality in common bean and promising molecular tools to aid future breeding efforts to answer consumers’ concerns.

## 1. Introduction

Consumers are increasingly more health-conscious and striving to have greater diversity and healthier foods in their diets [1]. Common bean, or bean (*Phaseolus vulgaris* L.), is an important and affordable source of protein, dietary fiber, essential vitamins, and minerals [2]. It is one of the most important food legumes cultivated and consumed worldwide [3], being a staple food in Eastern Africa and Latin America [4], and a component of the Mediterranean diet [5]. Consequently, bean is an important crop to fight malnourishment, particularly protein malnutrition [3], as well as an important food for the prevention of a variety of non-communicable diseases due to its diversity of health-promoting compounds, such as resistant starch and dietary fiber (reviewed in [6]). In addition to its nutritive composition, common bean can be consumed in a variety of culinary forms and specific high-quality landraces can attract high market prices (gourmet foods) [7].

Despite its compositional richness, common bean production and consumption have been declining in Europe. Several factors contributed to this, such as low productivity, changes in consumers’ preferences, and little investment in breeding and food innovation [8]. Nevertheless, breeding programs could be instrumental to reverse this decline through the development of bean varieties, first with higher and stable yields, increasing the attractiveness of this crop to farmers, but also with higher nutritional quality, more adapted to present consumers’ demands [8]. Although particularly important to increase bean consumption and market demand, the traits related to nutritional quality, like the contents of particular compounds, are normally complex, controlled by many genes with small effects, and highly influenced by environmental factors [9]. Consequently, nutritional quality is hard to handle by conventional plant breeding.

The complexity of food nutritional quality breeding comes also from potential hidden interactions among the quality-related compounds. In particular, legumes possess bioactive compounds which can act simultaneously as health-promoting compounds and anti-nutritional factors, impairing nutrients’ bioavailability, and influencing both taste and consumers’ food acceptability [10]. Protease inhibitors are among these bioactive compounds, as they are anti-inflammatory and anti-carcinogenic compounds, and simultaneously interfere with digestion through the irreversible inhibition of trypsin and chymotrypsin (reviewed in [6]). Trypsin inhibitors interfere for instance with protein digestibility, reducing protein quality, a nutritional quality-related trait that has gained importance in recent years [11]. Protein quality is also evaluated in terms of amino acid composition. Although considered valuable sources of protein, the nutritional quality of legumes protein is often lower than that of animal protein, due to their reduced content on sulfur amino acids (methionine and cysteine) [12]. Nevertheless, variation exists in nature or may be generated, and higher protein quality in terms of improved amino acid composition and digestibility is presently regarded as an important target for legume breeding [13].

A common aspect of the mentioned nutritional quality-related targets is that many of these nutritional compounds’ contents are laborious and expensive to measure and so difficult to routinely implement in breeding programs. However, the use of genomics-assisted breeding allows a considerable time and cost reduction in the development of crop varieties with improved nutritional contents as compared to conventional breeding [9]. Nevertheless, genomics-assisted breeding requires the clarification of the genetic basis of the target traits to be applied. Until now, studies on the genetic control of common bean seed composition have mainly focused on specific minerals such as iron and zinc. These studies used linkage mapping approaches, resorting to segregating populations from controlled crosses (e.g., [14,15]), or genome-wide association study (GWAS) approaches (e.g., [16,17]) to understand the genetic basis of these traits. However, only a reduced number of linkage and association mapping studies focused on macronutrients and protein quality. Examples are the study of Casañas et al. [18] on the genetic basis of ash, dietary fiber, starch, and protein among other traits, using a recombinant inbred line (RIL) population to perform a linkage mapping approach, or the Katuuramu et al. [19] study, on the genetic basis of protein and mineral contents using an association mapping approach.

Genome-wide association studies (GWAS) using populations of unrelated individuals to examine associations between genotypic polymorphisms and phenotypes are presently regarded as a good alternative to linkage mapping approaches to identify quantitative trait loci (QTL) responsible for complex traits variation [20]. Coupled with other recent advancements, such as the sequencing of reference genomes (e.g., the common bean genome [21]) or high-throughput next-generation sequencing (NGS) approaches, GWAS uncovers functional loci/genes underlying the genetic variation of complex traits with higher mapping resolution and broader genetic basis [22]. 

More than five centuries of bean cultivation in Portugal have produced a very diverse germplasm. Indeed, Portugal is considered a secondary center of origin of common bean diversity [23]. This germplasm diversity is expressed at agronomic, nutritional, and molecular levels [24,25] and proofed ideal for association genetic studies such as the identification of fusarium wilt resistance-associated candidate genes, and the identification of SNP alleles and candidate genes affecting photosynthesis under contrasting water regimes [26,27].

The present study aims to identify the genomic regions and/or candidate genes associated with common bean nutritional composition (ash, carbohydrates, fat, fiber, moisture, protein, and resistant starch contents) and protein quality (amino acid contents and trypsin inhibitor activity) within a diverse Portuguese germplasm collection, using a GWAS approach. We will test SNP-trait associations using linear mixed models accounting for the genetic relatedness between accessions and compare the SNP-traits association profile under two contrasting environments. By incorporating a heat stress environment in the study, we will be able to assess QTL stability under an expected climate change scenario for future exploitation of potential genotype × environment (G × E) for local adaptation. This study will also be useful for the development of molecular tools to facilitate routine evaluations on nutritional composition and protein quality, increasing the efficiency of common bean breeding for improved nutritional quality. 

## 2. Results

The present study was carried out to clarify the genetic architecture of nutritional composition and protein quality-related traits in common beans by making use of the Portuguese germplasm natural variation. For that, previously collected data [25] on the nutritional composition and protein quality of a diverse collection of 106 Portuguese common bean accessions, cropped in two contrasting environments (Cabrela, central Portugal, with a mild climate, and Córdoba, southern Spain, a heat stress prone region), was complemented with the resistant starch quantification of the same samples prior to a genetic analysis through a genome-wide association study.

The total phenotypic data analyzed included nutritional composition related traits such as macronutrient contents (protein, carbohydrates (CH), fat, fiber, ash, moisture, and resistant starch (RS)), and protein quality-related traits such as amino acids contents (Ala-alanine; Arg-arginine; Asp-aspartic acid; Glu-glutamic acid; Gly-glycine; His-histidine; Ile-isoleucine; Leu-leucine; Lys-lysine; Met-methionine; Phe-phenylalanine; Pro-proline; Ser-serine; Thr-threonine; Tyr-tyrosine; Val-valine), and trypsin inhibitor activity (TIA). Traits regarding nutritional composition were measured in the two contrasting environments, whereas protein quality-related traits were only measured in the samples harvested in the most stressed environment, Córdoba.

This phenotypic data was then integrated with previously obtained single-nucleotide polymorphism (SNP)-based genotypic data (16,689 SNPs before quality control), screened through Illumina Infinium BARCBean6k_3 BeadChip^TM^ assay and DArTseq^TM^ analysis [26]. Genomic regions associated with the traits were highlighted, through GWAS, taking into consideration the population structure.

### 2.1. Phenotypic Trait Variation

Most nutritional composition-related traits showed a similar range of phenotypic variation between environments (Figure S1). Nevertheless, for all these traits, apart from fat, significant differences were detected between the two environments, and most traits showed higher coefficients of variation, or variability, in Córdoba than in Cabrela (Table 1). Fat and resistant starch showed the highest variability among the nutritional composition-related traits, in both environments.

Of the traits measured only in Córdoba, trypsin inhibitor activity showed the highest variability (29.6%). Among the amino acid contents, Methionine stood out showing the highest variability (21.2%). The remaining amino acid contents had all very similar coefficients of variation. The amino acid present, on average, in lower amounts was Methionine (1.06 g/100 g) and the one present in higher amounts was Glutamic acid (20.44 g/100 g) (Table S1).

**Figure 1:**
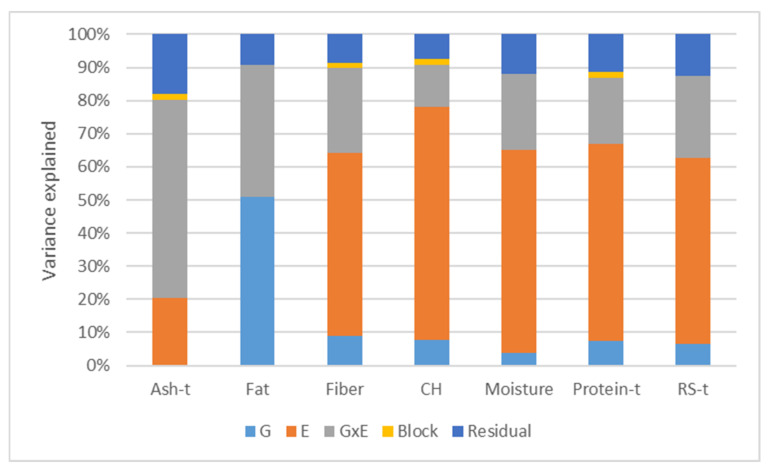
Variance components for the nutritional composition related traits measured in a collection of 106 Portuguese common bean accessions grown in two contrasting environments (Cabrela with a mild climate, and Córdoba, a heat stress prone region). The “-t” after the trait’s name indicates that data was transformed following a Box-Cox transformation. Genotype (G), environment (E), genotype by environment interaction (G×E), block, and residual (error), carbohydrates (CH), resistant starch (RS).

Variance components were estimated for the nutritional composition-related data obtained from the Córdoba and Cabrela trials taken together. A high influence of the environment (E) was observed for the majority of the traits (Figure 1). The effect of E ranged from 0 to 70.4% and the effect of G×E ranged from 12.8% to 60%. From the seven analyzed traits, ash and fat were the only ones that did not have the environment as the largest variance component. The biggest variance component for ash was G×E (60%), followed by E (20.3%), whereas the biggest variance component for fat was the genotype (G) (51%), followed by G×E (39.7%). Fat was thus an exception as the remaining six traits had variance components values inferior to 9% for G. Since the G×E effect was generally larger than G effects on the phenotypic variability, subsequent analyses were performed separately for the two contrasting environments with the exception of fat. 

Traits’ broad-sense heritabilities (Tables S2–S4) were, in general high, with values between 53% and 98%. The nutritional composition-related traits and trypsin inhibitor activity showed, in general, higher heritabilities than the amino acid contents. Also, the nutritional composition-related traits showed higher heritabilities for Córdoba than for Cabrela environment. Wald tests (Tables S2 and S3) indicated that there were significant differences among genotypes and no block effects for most of the measured traits. Wald test for fat (Table S4), calculated with genotype and environment terms fixed, indicated that there were significant differences among genotypes but not between environments.

A strong negative correlation between carbohydrates and protein (Pearson correlation coefficient, r = −0.97, Cabrela; r = −0.95, Córdoba; Figures S2 and S3), and a moderately strong negative correlation between carbohydrates and ash (r = −0.51, Cabrela; r = −0.63, Córdoba), were observed. Considerable positive correlations were identified between protein and ash (r = 0.62, Cabrela; r = 0.74 Córdoba) and between moisture and ash (r = 0.56, Cabrela; r = 0.40 Córdoba). There were strong positive correlations among the different amino acid contents, but only small correlations between these and the trypsin inhibitor activity. The correlations between protein quality-related traits and nutritional composition-related traits were, overall, small (Figure S2).

With the best linear unbiased estimators (BLUEs or adjusted means), two principal component analyses (PCAs) were computed: one with the nutritional composition data from both environments, and another with the nutritional composition and protein quality data measured only at Córdoba environment.

**Figure 2:**
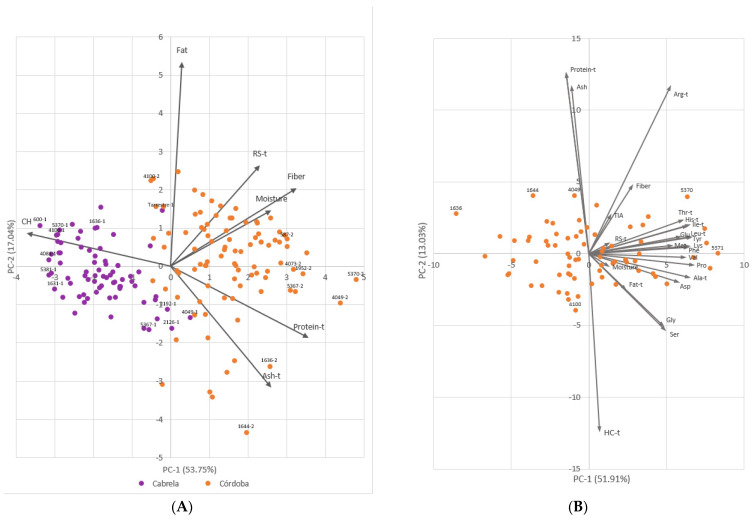
Principal component analysis based on BLUEs of nutritional composition and protein quality-related traits measured in a collection of 106 Portuguese common bean accessions. (**A**) Biplot representing accessions grown in Cabrela (purple) and accessions grown in Córdoba (orange). Trait loading vectors of the seven nutritional composition-related traits are represented by arrows. Relevant accessions are identified by their accession numbers followed by 1 or 2 according to the corresponding environment: 1-Cabrela; 2-Córdoba. (**B**) Biplot representing accessions grown in Córdoba (orange). Trait loading vectors of the 24 nutritional composition and protein quality-related traits are represented by arrows. CH-carbohydrates; RS-resistant starch; Ala-alanine; Arg-arginine; Asp-aspartic acid; Glu-glutamic acid; Gly-glycine; His-histidine; Ile-isoleucine; Leu-leucine; Lys-lysine; Met-methionine; Phe-phenylalanine; Pro-proline; Ser-serine; Thr-threonine; Tyr-tyrosine; Val-valine; TIA-trypsin inhibitor activity. The “-t” after the trait’s name indicates that data was transformed following a Box-Cox transformation.

The PCA with nutritional composition data collected in Cabrela and Córdoba environments (Figure 2A) depicted the impact of the environment on common beans’ nutritional composition as well as highlighted interesting quality accessions considering the measured traits. The two first principal components explained 70.79% of the total variability. The biplot demonstrates a clear separation of environments, with accessions collected in Córdoba showing higher variability and, in general, higher contents of ash, protein, moisture, fiber, and resistant starch. Examples of bean accessions grown in Córdoba with high contents of ash and protein are accessions 1636 and 1644. Complementing these, accessions 587, 1952, 4049, 4073, 5367, and 5370 showed some of the highest contents of protein and fiber in Córdoba but also high contents of ash and resistant starch in the same environment. Accession 4100 showed one of the highest values of fat, the highest value of carbohydrates in Córdoba, and one of the lowest values for protein, ash, fiber, and moisture. Interestingly, despite having the highest content of fiber among samples grown in Córdoba and one of the highest contents of protein, accession 5370 showed the opposite phenotype in the samples grown in Cabrela where it had one of the highest contents of carbohydrates. On the other hand, accessions 4049 and 5367 showed some of the highest contents of ash, protein, and fiber in Cabrela, maintaining a similar nutritional composition in Córdoba. Accessions 600, 1631, 4081, 4100, and 5381, grown in Cabrela, showed the highest contents of carbohydrates overall.

The second PCA (Figure 2B) included protein quality-related traits in addition to the nutritional composition traits measured in common bean accessions grown in the heat stress environment (Córdoba). The first two principal components explained 64.94% of the total variability, with the first principal component explaining most of the variability (51.91%). This PCA showed that accession 5370 was among the accessions with the highest protein contents and with the highest content of the various amino acids. On the other hand, accession 5371 had even higher amino acid contents, but only average contents of protein and remaining nutritional composition-related traits.

### 2.2. SNP-Trait Associations

**Figure 3:**
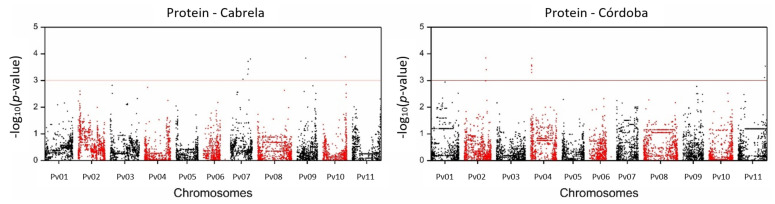
Manhattan plot depicting the genome-wide association results for protein content in common bean using 78 Portuguese accessions grown in the Cabrela environment (left) and 94 Portuguese accessions grown in the Córdoba environment (right). The y-axis represents the −log_10_ (*p*-value) of 9601 SNPs and the x-axis shows their chromosomal positions across the common bean genome. The horizontal red line indicates the significance threshold (*p*-value = 10^−3^).

Accessions adjusted means for each trait were tested for association with SNP data taking population structure or familial relatedness into consideration. Manhattan plots depicting GWAS results for protein are shown in Figure 3, all the remaining traits’ Manhattan plots are shown in Figures S4–S6. For most traits, the best model for association analysis, with an inflation factor closer to 1 (Table S5) and Q–Q plots showing fewer P-values deviating from the expected uniform distribution that holds under the null hypothesis (Figure S7), was the model using a different kinship matrix per chromosome. For His, Lys, Phe, Ser, and Thr a model using 15 principal components to control for population structure was used. 

A total of 224 marker-trait associations were identified for the 24 nutritional composition- and protein quality-related traits studied, with the defined threshold −log_10_ (*p*-value) = 3. The conservative Benjamini-Yekutieli *p*-value adjustment supported the significance of 151 (67%) of these associations (Table S6). Marker-trait associations were found for all traits except for Glu, Gly, His, Ile, Leu, Met, Pro, and Thr. A total of 181 unique SNPs was responsible for the 224 marker-trait detected associations, indicating that some of the SNPs were associated with more than one trait. The 181 associated SNPs were organized in 136 unique genomic regions, each genomic region englobing the markers within a linkage disequilibrium (LD) block. One hundred and five SNP markers were significantly associated with the nutritional composition traits measured in Cabrela and 37 in Córdoba, being one SNP marker (DART03724) commonly associated with the same trait (moisture) in both environments. Moisture was the trait with the biggest number of associations detected in one environment (62 in Cabrela but only 6 in Córdoba). However, a significant portion of the SNPs associated with moisture in Cabrela was located within the same LD blocks (62 SNPs were located within 36 genomic regions). Among protein quality-related traits, Arg content had the biggest number of associations detected (32). However, similarly to moisture, a large portion of these SNPs was within the same LD blocks (32 SNPs were located within 13 genomic regions). The association of the same SNP with different traits occurred mainly among amino acid content traits, being eight markers associated with more than one trait. SNP04308 and SNP09201 were the markers associated with the highest number of traits, each associated with four different amino acid contents. Except for Arg, out of the eight amino acid traits with associated markers, all shared markers with another trait.

Marker-trait associations were identified across all the common bean chromosomes. Amino acid contents, in particular, were mostly associated with markers belonging either to chromosome Pv02 or Pv09.

Most marker-trait associations explained only a small percentage of the observed phenotypic variance, with an average of 16.2%. Moisture (measured in Cabrela) was an exception to this, with markers explaining up to 70.6% (DART04597) of the observed phenotypic variance. Other SNPs explaining larger proportions of variance, besides the previously referred, were SNP01084 (45.6%; resistant starch, Cabrela), DART02462 (31.3%; fat, both environments), SNP01413 (29.7%; ash, Cabrela) DART11240 (24.0%; carbohydrates, Cabrela), and SNP00787 (24.1%; Arg, Córdoba) (Table S6). The variant allele for 64% of the associated SNP markers had a positive effect on the trait in this association panel.

### 2.3. Candidate Gene Identification

A gene was considered a putative candidate for the phenotypic trait under analysis if it contained an associated SNP or was in linkage disequilibrium (LD) with an SNP associated with the trait, observing a strict LD-decay threshold (r^2^ > 0.2). This was investigated using the JBrowse tool in *Phaseolus vulgaris* v2.1 genome in Phytozome v12 portal. One hundred and two different candidate genes were identified within 136 genomic regions responsible for trait variation. 

**Figure 4:**
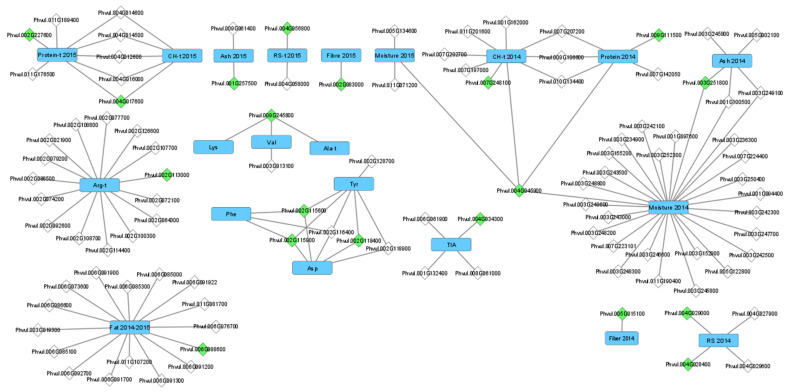
Network analysis of the candidate genes proposed for the nutritional quality traits using 106 Portuguese common bean accessions grown in two contrasting environments (Cabrela and Córdoba), using Cytoscape software. Traits represented as rectangles; genes represented as diamonds. Genes identified by green diamonds correspond to candidate genes for the SNP markers associated with the highest P-value for each trait. Traits are identified as: 2014-Cabrela; 2015-Córdoba; 2014–2015 — both environments; “-t” — trait data transformed following a Box-Cox transformation. CH-carbohydrates; RS-resistant starch; Ala-alanine; Arg-arginine; Asp-aspartic acid; Lys-lysine; Phe-phenylalanine; Tyr-tyrosine; Val−valine; TIA-trypsin inhibitor activity.

The gene-trait network (Figure 4) established for the 102 identified candidate genes showed a clear separation between groups of traits and environments. Candidate genes for protein quality-related traits did not connect to candidate genes for nutritional composition-related traits. Similarly, with one exception, candidate genes for traits measured in Cabrela did not connect to candidate genes for traits measured in Córdoba. The exception to this was gene Phvul.004G045900, encoding for a galacturonosyltransferase 9, which linked moisture measured in Córdoba to moisture, protein, and carbohydrates measured in Cabrela. Most connections of the same gene to various traits occurred among amino acids, with four different genes connecting three different amino acids. There were also various genes connected to both carbohydrates and protein, in both environments. 

**Figure 5:**
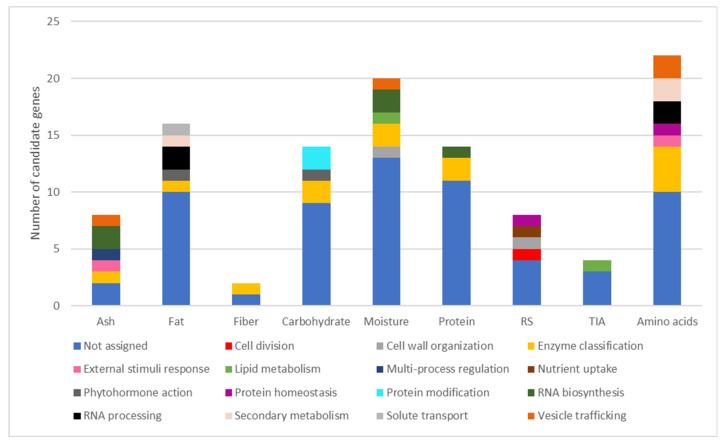
MapMan functional categories of the candidate genes associated with the nutritional composition and protein quality-related traits in 106 Portuguese common bean accessions grown in two contrasting environments. Candidate genes for amino acid contents were pooled together. The nine bar charts represent the number of candidate genes of a given functional category associated with ash, fat, fiber, carbohydrate, moisture, protein, resistant starch (RS), trypsin inhibitor activity (TIA), and amino acid contents.

Functional categorization of the candidate genes was obtained using MapMan (Mercator) web tools to better understand the involvement of the candidate genes in different metabolic pathways. From the candidate genes identified, 41.7% had a functional category assigned (Figure 5, Table S7). The assigned categories showed some diversity within each trait and a high diversity overall, with 15 different categories assigned to the 102 candidate genes. The most common functional categories attributed by MapMan across all traits were “enzyme classification” (12%), “RNA biosynthesis” (4.6%), “RNA processing” (3.7%), and “vesicle trafficking” (2.8%).

In the frame of this work, it was not possible to highlight all candidate genes located within the associated genomic regions in detail. We, therefore, restrict ourselves to highlight those that were (1) located within regions associated with multiple quality-related traits, and with (2) a biological annotation related to the studied trait. The candidate gene associated with the biggest number of quality related-traits, which also had a biological annotation related to the traits, was Phvul004G045900, encoding a galacturonosyltransferase 9, which was associated with carbohydrates, protein, and moisture (in both environments). Another candidate gene highlighted due to its association with more than one quality-related trait was Phvul010G13440, encoding a hydroxyproline-rich glycoprotein family protein, which was associated with protein and carbohydrate contents. Finally, the genes highlighted due to their biological annotation were Phvul004G056800, encoding an ankyrin repeat family protein, Phvul009G061400, encoding a cytochrome P450, family 82, subfamily C, polypeptide 4, and Phvul002G113000, encoding a transmembrane amino acid transporter family protein, respectively associated with resistant starch, ash, and Arg.

## 3. Discussion

This study explored the natural variation of 106 accessions from the highly diverse and underused Portuguese common bean germplasm grown under contrasting environments (traditional and heat stress), using a GWAS approach to unveil the genetic architecture of 24 nutritional compositions and protein quality-related traits. The generated knowledge will allow a better understanding of the molecular mechanisms and pathways regulating the nutritional composition and protein quality in common beans. Further, it will assist the development of promising molecular tools to help breeders answer consumers’ diet concerns and to support farmers to better exploit G×E quality interactions under future climate constraints. A total of 136 common bean genomic regions controlling the natural variation of the analyzed traits were identified in this association panel. Additionally, 102 putative candidate genes for the trait-associated regions were proposed.

### 3.1. Genomic Regions and Candidate Genes Associated with Common Bean Nutritional Composition and Protein Quality-Related Traits

SNP marker-trait associations were identified for all the nutritional composition and protein quality-related traits analyzed except for Glu, Gly, His, Ile, Leu, Met, Pro, and Thr. A mean of 9.5 marker-trait associations was identified per trait, each SNP explaining, on average, a small percentage of the phenotypic variation (around 16%). Mapping resolution to the gene level was achieved in 55.9% of the cases (LD blocks where a single gene was identified), demonstrating the complex genetic nature of common bean nutritional quality-related traits and validating the use of GWAS to harness the diversity of this Portuguese common bean germplasm.

The protein content is one of the most relevant food grain legume traits for breeding, as the interest in plant-based protein increases in developed countries to provide healthier diets, and the need for cheap protein sources to fight malnourishment in developing countries remains [28,29]. Previous studies identified Quantitative Trait Loci (QTL) for seed protein content on common bean chromosomes Pv05 and Pv07 using a Xana×Cornell 49242 RIL population, with parental lines belonging to the Andean and Mesoamerican gene pools, respectively [18], and on Pv03, Pv06, and Pv07 using a subset of the Andean Diversity Panel [19]. Our study identified significant marker-trait associations for protein content on chromosomes Pv02, Pv04, Pv07, Pv09, Pv10, and Pv11. The LD blocks around the five associated markers identified in Pv07 (DART06714, DART06845, DART06856, DART03216, and DART03273) were such that each marker was located in an independent genomic region. Of these five marker-trait associations one was at 23Mb, three between 32 and 33 Mb, and the fifth at 37 Mb. The QTL identified on Pv07 by Casañas et al. [18] was located 5Mb away from the QTL identified by Katuuramu et al. [19] which was located at 7.6 Mb. Therefore, both previously identified QTLs were located more than 10 Mb away from the presently identified QTLs, and therefore out of our LD windows. This suggests that in common bean several regions control seed protein concentration on chromosome Pv07. The different genotypic resources used between these three genetic studies may probably explain these findings. The use of association mapping populations, such as the one used in the present study, characterized by unrelated accessions of Andean, Mesoamerican, and of admixed genetic origin, or of Andean origin as in Katuuramu et al. [19], allows the exploration of a larger allelic diversity (broader genetic basis), with higher mapping resolution. This contrasts with the use of bi-parental linkage mapping populations which have a narrower genetic basis, resulting in a smaller potential identification of genomic regions associated with the trait of interest [22]. This might explain the smaller amount of genomic regions associated with protein content identified by Casañas et al. [18] using a RILs population developed from the cross of only two parental lines although from the different Andean and Mesoamerican gene pools.

Several of the markers associated with protein content were simultaneously associated with carbohydrate content (in both environments) but with contrasting effects on trait variation, reflecting the expected strong negative correlation between these related traits. One of these markers was SNP04726, which was the second most significantly associated with protein content (explaining 9.4% of the variability) and was also associated with carbohydrates content, explaining 10.8% of its variability (data from Cabrela field trial). A candidate gene including this marker sequence was Phvul.010G134400, which encodes a hydroxyproline-rich glycoprotein family protein (HRGP). HRGPs are known to accumulate in cell walls as a general response of dicotyledons to infection by biotrophic and necrotrophic fungi, bacteria, and viruses [30]. In particular, this reaction was described in common beans as a response to infection by the causal agent of anthracnose [31]. Selecting for this marker/gene candidate could allow an increase in protein content to the detriment of the carbohydrate content in new bean varieties. Nevertheless, several of the other markers associated with these traits in the present study were only associated with either protein or carbohydrate contents. Therefore, selecting for those markers could allow an increase or decrease of the contents of either trait (protein and carbohydrates) independently of the other trait.

Apart from the genes associated with both protein and carbohydrates contents, very few other SNPs/candidate genes related to nutritional composition were connected to more than one trait, reflecting the small correlations among most nutritional composition-related traits. Indeed, most SNPs and subsequent candidate genes for all the studied nutritional quality-related traits were only associated with one trait, as can be seen in the network analysis of candidate genes (Figure 4).

Of note, among the genes connected to more than one trait, is Phvul.004G045900, which encodes a galacturonosyltransferase 9. Two markers led to the identification of this gene, DART03724 which was associated with carbohydrate contents measured in seeds from Cabrela and moisture contents measured in both environments, and DART06845 which was associated with protein content measured in seeds from Cabrela. This gene was the only identified candidate associated with more than three traits, the only gene connected to the same trait in both environments, and with a corresponding variant allele responsible for the largest effect on trait variability for carbohydrates, protein, and moisture (data from Córdoba). Galacturonosyltransferases are required for the synthesis of pectin, a family of complex polysaccharides present in the cell walls of all land plants [32]. Moreover, pectin is involved in the regulation of ion transport and porosity of cell walls and is consequently involved in the control of cell wall permeability and determination of water holding capacity [33]. Therefore, galacturonosyltransferase 9 seems to be an interesting candidate for the variation of both carbohydrate and moisture contents in common bean seeds. 

Moisture measured in seeds from Cabrela showed the biggest number of marker-trait associations detected (62) and the largest percentage of variation explained by a marker (70.6%). However, a significant portion of the associated SNPs was located within the same LD blocks, as 62 SNPs were located within 36 genomic regions. Two chromosomes, Pv07 and Pv03, are of particular interest and could be responsible for most of the observed variation. Pv07 contains one genomic region which includes the marker responsible for 70.6% of the moisture phenotypic variation. Pv03 contains most of the markers associated with moisture (43) as well as the marker associated with this trait at the highest statistical significance. Most of the associated markers within this chromosome (37) were enclosed within a relatively small section of the chromosome (3Mb) and were thus relatively close physically. Despite the physical closeness, 25 genomic regions (LD blocks) were still detected within this section of the chromosome suggesting that various QTL may be responsible for the phenotypic variation of moisture within this chromosomal region. Moisture from Cabrela contrasted with most other traits in the amount of explained variability per QTL, including moisture measured in seeds from Córdoba. In the Córdoba environment, moisture was associated with six markers, each explaining less than 9% of the phenotypic variance. The difference in the proportion of variance explained between environments was mainly due to higher effects of each marker on the phenotypic variation in Cabrela (up to 0.7) than in Córdoba (up to 0.3). This difference might indicate some inflation of the effect attributed to each marker. This inflation could be explained by the relatively small mapping population used since smaller populations lead to inflated QTL effects [34].

QTLs for ash, fiber, and starch in common bean were also identified through linkage mapping in the previously mentioned study of Casañas et al. [18]. In that study, QTLs for ash were identified on chromosomes Pv01 and Pv07, for fiber on Pv06 and Pv07, and for starch on Pv01, Pv02, Pv04, and Pv07. Similarly, our study identified a marker-trait association for ash on chromosome Pv01, and for resistant starch on Pv01 and Pv04. A candidate gene for DART03741, associated with resistant starch on Pv04, is Phvul.004G056800, encoding an Ankyrin repeat family protein. This family of proteins includes members implicated in carbohydrate allocation and has been associated with reduced starch in tobacco transformants carrying a specific ankyrin repeat family gene [35].

Also associated with ash content was the SNP03984 in Pv09. A candidate gene for this associated SNP is Phvul.009G061400 which encodes a cytochrome 450, family 82, subfamily C, polypeptide 4 (CYP82C4). CYP82C4 is a heme-containing enzyme that is strongly correlated with genes involved in metal uptake/transport and is possibly involved in the early iron deficiency responses in *Arabidopsis thaliana*, as was proposed by Murgia et al. [36]. The involvement of this gene in the uptake/transport of iron becomes particularly interesting when considering that iron deficiency is the most common and widespread nutritional disorder in the world [36].

The strong correlations among amino acid contents were reflected by the association of several SNPs to more than one of these traits. As an example, four molecular markers and the respective candidate genes were associated with three different amino acid contents: DART01478, SNP00739, SNP00741 were associated with Asp, Phe and Tyr; and SNP04308 was associated with Ala, Lys, and Val contents. Interestingly, Arg was the only amino acid content that did not share SNPs and subsequently candidate genes with other amino acid contents. Of the several SNP markers associated with Arg, the one within the candidate gene Phvul.002G113000 was the most strongly associated and the one that explained the highest proportion of variance (20.2%). This gene encodes a protein of the transmembrane amino acid transporter superfamily. These are integral membrane proteins involved in the absorption of amino acids from the soil, load and transport of amino acids in the phloem, absorption of amino acids in seeds, and long-distance transport and distribution of amino acids in seeds [37]. Functional analysis of this gene would be relevant to understand the usefulness of this gene and whether the content of Arg is the only affected by this variant allele, as the marker has not been considered as associated with the other amino acid contents due to the chosen −log_10_ (P) threshold.

### 3.2. Portuguese Common Bean Germplasm Nutritional Quality Richness and the Influence of Environment

As has been previously shown, the environment, in particular heat and drought stress, influence the maturing process of seeds, affecting various nutritional quality traits in the process (reviewed in [38]). Accordingly, environment and G×E had large effects on the nutritional quality traits variation observed in the Portuguese common bean germplasm collection and scored across field trials. Common bean accessions grown in Córdoba (heat stress environment) showed higher variability, as well as higher average contents of ash, fiber, moisture, protein, and resistant starch than accessions grown in Cabrela (milder environment). On the other hand, carbohydrates and moisture contents were on average lower in Córdoba than in Cabrela. As discussed by Mecha et al. [25], despite the general connection between reduced yield and heat stress [39], no differences in yield were observed in the two environments used in this study, likely due to the presence of artificial irrigation. Therefore, the differences in nutritional composition observed between environments were likely due to heat stress. In particular, heat stress has been associated with alterations in carbohydrate metabolism, affecting synthesis but also the accumulation of carbohydrates during seed filling [38,40], which could explain the reduction of carbohydrate contents in the seeds grown in Córdoba. Unlike the remaining nutritional composition traits, fat was the only trait with a reduced effect of the environment on trait variability.

In addition, G×E was also significant on the traits analysed in both environments, particularly for ash and fat contents. G×E occurs when genotypes differ in their relative performance across environments and corresponds to the presence of genetic factors with environment-specific effects [41]. The magnitude of G×E varies among traits and accessions and in some cases can lead to crossover interactions as can be seen in the present study with accession 5370, for example, which showed one of the highest contents of protein when grown in Córdoba and one of the lowest contents of protein when grown in Cabrela. Another example is accession 1636, which showed one of the highest contents of fat when grown in Cabrela and lowest contents of fat in Córdoba, and the opposite concerning ash (one of the highest contents of ash in Córdoba and one of the lowest in Cabrela). In other cases, crossover interactions do not occur, and the relative performance of the genotypes is maintained across environments, as can be seen with accession 4049, for example, which showed one of the highest contents of protein, ash, and fiber in both environments.

As previously stated, DART03724 with Phvul.004G045900 as a putative candidate gene, was the only molecular marker/gene associated with the same trait (moisture) in both environments. The lack of more markers stably associated with traits across environments is a consequence of the environment-specific nature of the genomic regions associated with the studied traits. Constitutive QTLs, which show consistent effects across environments, are the main targets for breeding programs as they can improve crop performance across various regions where the crop can be grown. Nevertheless, it is possible to take advantage of significant G×E by breeding for local adaptation [41] and also increase the efficiency of this selection using the specifically associated molecular markers instead of performing time-consuming and expensive phenotyping. Examples of promising associated markers explaining considerable amounts of trait variability are DART11240 for carbohydrates content (in Cabrela) and SNP00732 for Arg (in Córdoba) that explained 24.0% and 20.2% of these traits’ phenotypic variation. The fact that the great majority of the presently identified QTLs are environment-specific indicates that breeding efforts for nutritional quality traits in the Portuguese common bean collection should focus on developing varieties adapted to location-specific growing conditions, as proposed by Vaz Patto and Araújo [8]. The higher variability within traits observed in the stressed environment of Córdoba highlights the genetic richness of the Portuguese germplasm collection and its potential for location-specific breeding, namely for production under heat stress conditions.

Under these more stressful conditions, particular Portuguese accessions stood out as promising sources of protein quality-related traits. Traits such as bioactive compounds that influence protein digestibility and limiting amino acids are factors used to determine protein quality [42]. Limiting amino acids are the essential amino acids, or the amino acids that must necessarily be provided by the diet, that are in shortest supply [6]. As expected in a legume species, Met was the limiting amino acid (1.06 g/100 g) in the Portuguese common bean accessions analyzed. Pearson correlations showed that amino acid contents were strongly correlated among themselves and weakly correlated with TIA and the nutritional composition traits. As a consequence of the strong correlation among amino acids contents, targeted conventional breeding for increased Met should not be feasible. This can be explained by the low Met content of phaseolin, the main storage protein of common bean (40–50% of the total protein content), which despite being deficient in Met is still the major source of this amino acid [43]. Montoya et al. [43] proposed instead the exploration of the natural variability of phaseolin in terms of their protein digestibility, to increase the availability of Met and improve the protein quality of common bean. Although highly reduced by the processing, namely boiling (reviewed in [42,44]), the presence of trypsin inhibitors compromises protein digestibility and amino acid absorption, thus affecting protein quality (reviewed in [6]). In general, the accessions that showed in the present study the highest contents of protein and amino acids were also the accessions with the highest TIA. For example, accession 5371 displayed one of the highest contents of amino acids but also high contents of TIA. However, the reduced correlation of TIA with the remaining protein quality-related traits at the genetic level (different genomic regions involved in their control) suggests the possibility of altering the contents of amino acids and TIA independently through conventional breeding, which is especially facilitated by the help of the detected associated molecular markers.

Due to the detailed and expensive nature of the analysis developed to measure some of the nutritional quality traits, the size of the population and the number of tested environments had to be restricted in the present study. Smaller populations lead to lower QTL detection power as well as inflated estimates of QTL effects [34]. Nevertheless, the relatively high heritability observed in the analyzed traits compensates for the small population size to some extent, as the power for detecting QTLs is a function of population size x heritability of the trait [34]. Nevertheless, we were able to detect the most relevant QTL for breeding in this association panel, as QTL with minor effects are the ones harder to detect [34]. Additionally, due to the previously referred constraints, protein quality-related traits were only measured in one of the environments. This decision was based on the higher variability observed in that environment but allowed us also to collect phenotypic data in the heat stress environment mimicking future climate changes expected in the Mediterranean region.

In conclusion, this study further characterized the Portuguese common bean germplasm through the clarification of its genetic architecture of nutritional quality traits. The functional categorization of the proposed 102 candidate genes for 24 nutritional composition and protein quality-related traits demonstrated the involvement of a variety of metabolic pathways in the determination of common bean nutritional quality, corroborating the genetic complexity of these traits. Additionally, this study provided a unique resource of molecular markers associated with common bean nutritional and protein quality traits, which will help to answer consumers’ nutritional demands as well as broader vs. local adaptation on future breeding efforts. In particular, the inclusion of data from Córdoba, a heat stress environment, allowed the identification of markers relevant for quality breeding in a context of climate change with increasing temperature scenarios, such as the one being experienced in the Mediterranean area.

## 4. Materials and Methods

### 4.1. Plant Material and Growing Conditions

A collection of 106 common bean accessions, from the Portuguese plant germplasm bank (BPGV, INIAV, Braga, Portugal) was used for the present genetic study. This collection was the same as described by Leitão et al. [26,27] to identify genomic regions controlling fusarium wilt resistance and photosynthetic efficiency-related traits in common bean. Based on genotypic data, it is known that this collection is mainly composed of accessions belonging to the Andean gene pool and a smaller proportion to the Mesoamerican gene pool. Additionally, one-third of the accessions have an admixed origin and might represent putative hybrids between the Andean and Mesoamerican gene pools [24].

Seeds from the bean accessions were sown in two different environments following a randomized complete block design, with two replicates at each environment, as described by Mecha et al. [25]. The field trials were developed in two different years and locations. The first field trial took place from May to September 2014 in Cabrela, Portugal, and the second from March to July 2015 in Córdoba, Spain. Cabrela represents a standard common bean production area in Portugal, characterized by temperature average ranges of 18–21 °C and 66–80% of relative humidity during the growing season, whereas Córdoba represents a heat stress prone area, characterized by temperature average ranges of 15–32 °C and 31–63% of relative humidity during the growing season. The two field trials were established under artificial irrigation. Mature dried seeds were collected from a total of 106 accessions, 66 in both environments, 12 exclusively in Cabrela (environment with a total of 78 accessions) and 28 exclusively in Córdoba (environment with a total of 94 accessions). The mature dried seeds were milled to a particle size of 0.8 mm and stored at −20 °C until chemical analysis.

### 4.2. Phenotypic Data Acquisition

Twenty-three traits related to nutritional composition and protein quality were measured in the common bean harvested from the two field trials as described by Mecha et al. [25] and retrieved for the present genetic association study. Total protein, total carbohydrates (CH), fat, fiber, moisture and ash contents, were measured in the samples from Córdoba and Cabrela, and the contents of 16 different amino acids (Ala-alanine; Arg-arginine; Asp-spartic acid; Glu-glutamic acid; Gly-glycine; His-histidine; Ile-isoleucine; Leu-leucine; Lys-lysine; Met-methionine; Phe-phenylalanine; Pro-proline; Ser-serine; Thr-threonine; Tyr-tyrosine; Val-valine) and trypsin inhibitor activity (TIA), were measured only in the samples from the most stressful environment (Córdoba). In addition, in the present study, resistant starch (RS) content was measured in all the harvested samples, completing a total of 24 traits analyzed.

Briefly, Mecha et al. [25] determined total protein, fat, fiber, moisture and ash (%) content using a near-infrared (NIR) analyzer (MPA; Bruker, Billerica, MA, USA). Total carbohydrates were calculated following Equation (1):

total carbohydrates = 100 − (total protein + total fat + moisture + ash).(1)

A LC-MS/MS system Waters Alliance 2695 HPLC system coupled to a triple quadrupole mass spectrometer, Micromass® Quattro micro API (Micromass, Waters, Milford, MA, USA), equipped with an electrospray ionization source (ESI) was used to determine the content of the 16 amino acids [25]. Protein was hydrolyzed using a solution of HCL 6 M with 0.1% of phenol and the final amino acid extract resulted from the resuspension of the hydrolysates in HCL 0.1M after the evaporation to dryness of the initial solution.

The chromatographic separation was performed in a Mediterranean Sea 18, 5 µm 20 × 0.21 cm, 1.8 µm, (Teknokroma®, Barcelona, Spain) column. The amino acids were analyzed by multiple reaction monitoring (MRM) mode, using an ESI source operating in ion positive mode. Amino acids were identified by comparison with the amino acids’ standard retention time and corresponding m/z values.

Trypsin inhibitors were extracted from 0.5 g of common bean flour to which 25 mL of NaOH 0.01M were added, and the pH was adjusted to 9.5 ŷ 0.1. Inhibition percentage and trypsin inhibitor activity were calculated according to Mecha et al. [25]. Resistant starch was quantified following the methods AACC 32-40.01 [45] and AOAC 2002.02 [46]. The method was performed using a Resistant Starch Assay Kit (K-RSTAR, Megazyme, Bray, Ireland). A buffer solution of sodium maleate (pH 6.0), containing pancreatic α-amylase and amyloglucosidase, was added to the thawed samples of common bean flour. The samples were incubated in a water bath with horizontal agitation (100 rpm) at 37 °C for 36 h. The reaction was interrupted with the addition of ethanol (99%). The solution was centrifuged at 3000 rpm for 10 min, and the pellet, containing resistant starch, was washed two additional times with ethanol (50%) followed by centrifugation. The resulting pellet was resuspended in potassium hydroxide (2M) under continuous agitation in an ice and water bath for 20 min. The solution was then neutralized with a buffer solution of sodium acetate (pH 3.8). The existing starch was hydrolyzed to glucose through the action of the amyloglucosidase in a water bath at 50 °C for 30 min. The samples were centrifugated, and two 0.1 mL aliquots were collected from the liquid phase. Simultaneously, a blank sample was prepared with 0.1 mL of sodium acetate 0.1 M (pH 4.5) as well as four standard glucose solutions with 0.1 mL of glucose solution (1 mg/mL). For glucose quantification, through spectrophotometry, 3 mL of glucose oxidase/peroxidase reagent were added to each tube, followed by a 50 °C incubation for 20 min. The absorbance of the samples was evaluated at 510 nm against the blank sample. The average glucose content of each sample was compared to the absorbance values of the standard glucose solutions to obtain the concentration of resistant starch.

### 4.3. Phenotypic Data Analysis

Quality control of phenotypic data was performed separately for each environment. Technical repetitions were averaged for each accession to minimize technical error. A descriptive statistical analysis was performed using the summary statistics option of Genstat® software, 21st edition [47]. Histograms and boxplots were generated per trait to analyze data distribution and to identify outliers. The normality of residuals was assessed for each trait using the Shapiro-Wilk test. A Box-Cox transformation was applied when needed to meet normality assumptions.

A linear mixed model was fitted per trait as trait = genotype + block + error for the analysis of traits measured in a single environment and as trait = genotype + environment + genotype x environment + block + error for the analysis of traits measured in both environments using the restricted maximum likelihood (REML) variance component analysis framework of Genstat software, where environment identifies the two field trials and block identifies the two plot replicates within each trial.

Models were initially fitted with all terms as random to obtain the best linear unbiased predictors (BLUPs), estimate variance components, broad-sense heritability and Pearson correlation coefficients between traits. In a second step, genotype and block were fitted as fixed terms to obtain the best linear unbiased estimates (BLUEs), for each trait and accession. BLUEs were determined for fat with genotype and environment fitted as fixed terms. Wald tests for the significance of fixed effects were performed. BLUEs were used for principal component analysis (PCA) and as input phenotypic data for GWAS.

To estimate how much of the variation of accessions’ nutritional composition could be explained by the environment or the interaction with the environment, the previously defined model was fitted considering environment and genotype × environment as fixed terms, and a Wald test was performed to test for the significance of the fixed effects.

### 4.4. Genotypic Data

#### 4.4.1. Association-Mapping Analysis

Genome-wide association studies were conducted for all the 24 traits using the QTL library procedures from Genstat software. Adjusted means (BLUEs) for each trait were tested for association with a previously collected genotypic dataset [26] retrieved for the present study. This genotypic dataset was constituted of 9601 single nucleotide polymorphisms (SNPs) after quality control (removal of SNP markers and accessions with >25% missing data, as well as SNPs with a minor allele frequency <0.01 from the 16,689 SNPs originally screened) and was obtained through two different approaches, the Illumina Infinium BARCBean6k_3 BeadChipTM assay and DArTseqTM analysis [26]. SNPs called heterozygous were set as missing data. Furthermore, some accessions were not phenotyped for some of the traits. Thus, association mapping was performed using 72 accessions for the amino acid contents and trypsin inhibitor activity and 94 accessions for the remaining traits measured in seeds from Córdoba; and for 78 accessions for all the traits measured in Cabrela.

GWAS was performed separately for Cabrela and Córdoba environments for the traits that showed a higher variance component of genotype by environment (GxE) interaction than of genotype (G). Otherwise, the association study was performed with data from both environments together. GWAS was performed in the mixed model framework of Genstat software, fitting SNP as fixed and genotype as random terms using REML [48]. Four models were tested to detect significant marker-trait association: a null model [Phenotype = SNP + Error], which does not account for any population structure or familial relatedness; a model accounting for population structure (Q) [Phenotype = Q + SNP + Error], using 15 principal components from the principal component analysis (PCA); and two models accounting for familial relatedness (K) [Phenotype = SNP + genotype + Error], one with genotype random effects structured following a kinship matrix K [48,49]; and another using a different kinship matrix calculated for each chromosome using only the SNPs located on the remaining 10 chromosomes, as proposed by Cheng et al. [50]. The kinship matrices to account for familial relatedness per chromosome among genotypes were previously calculated by Leitão et al. [26] and retrieved to perform the present association studies. Inflation factor values near 1 and quantile-quantile (Q–Q) plots of the respective *p*-values with lower deviations from the expected uniform distribution under the null hypothesis were the considered parameters to select the best model accounting for genetic structure/relatedness among genotypes.

The observed −log_10_ (*p*-value) of each SNP was plotted against its chromosomal position to produce a Manhattan plot. Significant SNP-trait associations were detected at a threshold of −log_10_ (*p*-value) = 3. This threshold was set taking into consideration two aspects: the size of the association panel used and the background noise of the obtained Manhattan plots. Similar criteria were already described in other works with comparable panel sizes and a similar number of markers, focusing on resistance/tolerance traits [26] to avoid losing potentially interesting regions while applying a conservative type of adjustment such as Bonferroni correction. However, as a “conservative” guidance, adjusted *p*-values following the Benjamini and Yekutieli (BY) false discovery rate (FDR) method [51] were calculated, with α = 0.2 and k = 520 (the effective number of independent tests was set as the number of LD blocks per chromosome [52]), to control type I errors due to multiple testing.

For every SNP significantly associated with a trait, the effect of the minor-frequency SNP variant was calculated. The proportion of variance explained by each SNP-trait association was estimated using the formula V_QTL_/V_pheno_, where V_QTL_ = 2freq(1-freq)effect^2^ and V_pheno_ is the phenotypic variance of the adjusted means of each trait [53].

#### 4.4.2. Candidate Gene Identification

A gene was considered a putative candidate for the phenotypic trait under analysis if it contained an associated SNP or was in linkage disequilibrium (LD) with an associated SNP observing a strict LD-decay threshold (r^2^ > 0.2). LD was previously calculated for each common bean chromosome using the squared coefficient of the correlation between marker pairs r^2^ [26], and retrieved for the present study. Neighbouring SNPs showing r^2^ > 0.2 in relation to the associated SNPs were considered to be within the same LD block or genomic region. Putative candidate genes were searched for using the JBrowse tool in the *Phaseolus vulgaris* v2.1 genome (DOE-JGI and USDA-NIFA, http://phytozome.jgi.doe.gov/, accessed on 16 December 2021), available at the Phytozome v12 portal [54]. The annotation of the candidate genes was obtained from the file “Pvulgaris_442_v2.1.annotation_info.txt”, available in the previously referred portal.

Candidate genes were also assigned to MapMan bins, which described biological contexts/concepts, using Mercator4 V2.0 [55]. Cytoscape software [56], version 3.8.2, was used to visualize the candidate genes associated with each trait as a network.

## Figures and Tables

**Table 1:** Average ± standard deviation (and coefficient of variation (%)) of ash, fat, fiber, carbohydrates (CH), protein, moisture, and resistant starch (RS) contents (g/100 g) in a collection of 106 Portuguese common bean accessions grown in two contrasting environments (Cabrela with a mild climate, and Córdoba, a heat stress prone region). Data calculated from best linear unbiased estimators (BLUEs). In each column different letters indicate significant differences (*p* < 0.05).

	Ash	Fat	Fiber	CH	Moisture	Protein	RS
Cabrela	3.16 ± 0.08 ^a^ (2.6)	1.44 ± 0.24 ^a^ (16.4)	5.75 ± 0.45 ^a^ (7.8)	60.57 ± 1.53 ^a^ (2.5)	13.55 ± 0.47 ^a^ (3.5)	21.28 ± 1.44 ^a^ (6.8)	30.74 ± 3.49 ^a^ (11.4)
Córdoba	3.25 ± 0.13 ^b^ (4.1)	1.49 ± 0.32 ^a^ (21.7)	6.77 ± 0.72 ^b^ (10.7)	56.65 ± 1.75 ^b^ (3.1)	14.48 ± 0.51 ^b^ (3.5)	24.14 ± 1.66 ^b^ (6.9)	45.23 ± 11.36 ^b^ (25.1)

## Data Availability

Data is contained within the article or Supplementary Material.

## Supplementary Materials

The following are available online at https://www.mdpi.com/article/10.3390/plants11010026/s1, Figure S1: Histograms of the 24 nutritional composition and protein quality traits measured in a collection of 106 Portuguese common bean accessions. Figure S2: Pearson’s correlations between the 24 nutritional composition and protein quality traits measured in the seeds of a collection of 72 Portuguese common bean accessions grown in the Córdoba environment. Figure S3: Pearson’s correlations between the seven nutritional composition traits measured in the seeds of a collection of 78 Portuguese common bean accessions grown in the Cabrela environment. Figure S4: Manhattan plot depicting the genome-wide association results for ash, fiber, carbohydrates, moisture, and resistant starch content in common bean using 78 Portuguese accessions grown in the Cabrela environment. Figure S5: Manhattan plot depicting the genome-wide association results for ash, fiber, carbohydrates, moisture, protein, resistant starch, Alanine, Arginine, Aspartic acid, Glutamic acid, Glycine, Histidine, Isoleucine, Leucine, Lysine, Methionine, Phenylalanine, Proline, Serine, Threonine, Tyrosine, Valine and Trypsin inhibitor activity content in common bean using 94 Portuguese accessions grown in the Córdoba environment. Figure S6: Manhattan plot depicting the genome-wide association results for fat content in common bean using 106 Portuguese accessions grown in the Cabrela and Córdoba environments. Figure S7: Quantile-quantile (Q-Q) plots for the SNP-trait associations of the 24 nutritional composition and protein quality-related traits measured in Cabrela, Córdoba, or both environments. Table S1: Average, standard deviation, and coefficient of variation (%) of 16 amino acids and trypsin inhibitor activity (g/100 g) measured in the seeds of 72 Portuguese common bean accessions grown in Córdoba. Table S2: Wald test statistics and broad-sense heritability for several nutritional composition traits measured in the seeds of 78 Portuguese common bean accessions grown in the Cabrela environment. Table S3: Wald test statistics and broad sense heritability for several nutritional composition and protein quality traits measured in the seed of 94 Portuguese common bean accessions grown in the Córdoba environment. Table S4: Wald test statistics and broad sense heritability for fat measured in the seed of 106 Portuguese common bean accessions grown in two contrasting environments. Table S5: Inflation factors for the linear mixed models tested for genome-wide association of 24 nutritional composition and protein quality-related traits measured in common bean seeds collected in Cabrela and Córdoba. Table S6: SNP associations (−log_10_ (*p*-value) ≥ 3) with 24 nutritional composition and protein quality traits under two contrasting environments (Cabrela and Córdoba), marker position within chromosomes, allelic reference and allelic variant for the associated SNP, minor allele frequency, the effect of the allelic variant (rare allele), and the proportion of phenotypic variance explained by each associated SNP detected using a panel of 106 Portuguese common bean accessions. Table S7: Putative candidate genes for the 16 nutritional composition and protein quality traits for the significant (−log_10_ (*p*-value) ≥ 3) SNP-trait associations under two contrasting environments (Cabrela and Córdoba).
