# Supplementary material for: Portuguese Common Bean Natural Variation Helps to Clarify the Genetic Architecture of the Legume’s Nutritional Composition and Protein Quality
Source: Plants (Basel). 2021 Dec 22;11(1):26. doi: 10.3390/plants11010026 (PMC8747538; doi:10.3390/plants11010026)
Supplement: Supplementary file 1 [file plants-11-00026-s001.zip › plants-1508998-supplementary.pdf]

Supplementary Tables

Table S1. Average, standard deviation, and coefficient of variation (%) of 16 amino acids and trypsin inhibitor activity (g/100g) measured in the seeds of 72 Portuguese common bean accessions grown in Córdoba.

	Mean	Standard deviation	Coefficient of variation (%)
Ala	4.56	0.52	11.5
Arg	4.49	0.62	13.9
Asp	10.18	1.24	12.2
Glu	20.44	2.44	11.9
Gly	4.54	0.53	11.7
His	2.88	0.37	12.8
Ile	4.71	0.57	12
Leu	8.29	0.98	11.8
Lys	6.4	0.7	10.9
Met	1.06	0.23	21.2
Phe	5.94	0.59	9.9
Pro	3.95	0.43	10.9
Ser	5.42	0.79	14.5
Thr	3.62	0.54	15
Tyr	3.48	0.45	12.9
Val	5.11	0.54	10.6
TIA	8.96	2.65	29.6

Ala–alanine; Arg–arginine; Asp–aspartic acid; Glu–glutamic acid; Gly-glycine; His–histidine; Ile–isoleucine; Leu–leucine; Lys–lysine; Met–methionine; Phe–phenylalanine; Pro–proline; Ser–serine; Thr–threonine; Tyr–tyrosine; Val–valine; TIA - trypsin inhibitor activity.

Table S2. Wald test statistics and broad-sense heritability for several nutritional composition traits measured in the seeds of 78 Portuguese common bean accessions grown in the Cabrela environment. Acc-Accession. CH-carbohydrates; RS-resistant starch; "-t" trait data transformed following a Box-Cox transformation.

	**Wald Statistics**		**Heritability (%)**
Trait	Acc	Block	
Ash	<0.001	0.13	77.05
Fiber	<0.001	0.20	81.24
CH-t	<0.001	0.44	81.39
Moisture	<0.001	0.42	70.66
Protein	<0.001	0.42	80.49
RS	<0.001	0.83	66.90

Table S3. Wald test statistics and broad sense heritability for several nutritional composition and protein quality traits measured in the seed of 94 Portuguese common bean accessions grown in the Córdoba environment. The "-t" after the trait's name indicates that data was transformed following a Box-Cox transformation.

	**Wald Statistics**		**Heritability (%)**
Trait	Acc	Block	
Ash	<0.001	<0.001	88.93
Fiber	<0.001	<0.001	89.61
CH-t	<0.001	<0.001	82.41
Moisture	<0.001	0.128	88.11
Protein-t	<0.001	<0.001	81.45
RS-t	<0.001	0.575	85.23
Ala-t	<0.001	0.168	64.66
Arg-t	<0.001	0.139	61.17

Asp	<0.001	0.998	66.31
Glu	<0.001	0.836	64.75
Gly	<0.001	0.174	62.79
His-t	<0.001	0.83	61.81
Ile-t	<0.001	0.881	58.9
Leu-t	<0.001	0.363	58.84
Lys	<0.001	0.985	62.29
Met	<0.001	0.676	63.26
Phe	<0.001	0.486	53.44
Pro	<0.001	0.053	64.31
Ser	<0.001	0.143	70.31
Thr-t	<0.001	0.755	67.83
Tyr	<0.001	0.52	64.55
Val	<0.001	0.327	56.61
TIA	<0.001	0.397	75.15

CH—carbohydrates; RS—resistant starch; Ala—alanine; Arg—arginine; Asp—aspartic acid; Glu—glutamic acid; Gly—glycine; His—histidine; Ile—isoleucine; Leu—leucine; Lys—lysine; Met—methionine; Phe—phenylalanine; Pro—proline; Ser—serine; Thr—threonine; Tyr—tyrosine; Val—valine; TIA—trypsin inhibitor activity.

Table S4. Wald test statistics and broad sense heritability for fat measured in the seed of 106 Portuguese common bean accessions grown in two contrasting environments (Cabrela and Córdoba). Acc (accession), Env (environment).

	**Wald Statistics**		**Heritability (%)**
Trait	Acc	Env	
Fat	<0.001	0.781	63.18

Table S5. Inflation factors for the linear mixed models tested for genome-wide association of 24 nutritional composition and protein quality-related traits measured in common bean seeds collected in Cabrela and Córdoba, using 9601 SNPs. The values for kinship per chromosome are an average of the inflation factors for each chromosome. Blank spaces are the result of runtime errors during the GWAS analysis following the respective model for genetic relatedness correction. The "−t" after the trait's name indicates that data was transformed following a Box-Cox transformation.

**Cabrela**				
	Kinship [a]	Eigenanalysis [b]	Kinship per chr [c]	Null [d]
Ash	1.0830	1.2990	0.8919	1.2990
Fibre	0.9439	0.7492	0.8968	0.7492
CH-t	0.7145	0.6933	0.8385	0.6933
Moisture	2.3970	2.4110	0.9058	1.9030
Protein	0.8853	0.8981	0.8861	0.8981
RS	0.9539	0.9255	0.9094	0.9255
Córdoba				
	Kinship [a]	Eigenanalysis [b]	Kinship per chr [c]	Null [d]
Ala-t	2.3840	0.9458	0.8853	0.9458
Arg-t	2.2580	2.1290	0.9453	2.1290
Ash	1.5400	1.8150	0.8778	2.4960
Asp	1.1690	1.0400	0.9201	1.0400
Fibre	1.9820	1.2430	0.8844	1.2430
Glu	0.8013	0.9065	0.9496	0.9065
Gly	1.0080	0.7879	0.8391	0.7879
CH-t	2.1290	2.2260	0.8705	3.0280
His-t	1.3380	0.6342	-	0.6342
Ile-t	2.6750	0.8672	0.8271	0.8674
Leu-t	3.6000	0.9889	0.9239	0.9889
Lys	-	0.7492	-	0.7492
Met	-	0.7882	0.8828	0.7882

Moisture	0.5012	0.5776	0.8705	0.8072
Phe	1.0920	0.8007	-	0.8007
Pro	-	0.8240	0.8137	0.8240
Protein-t	1.9460	2.1170	0.9440	2.9970
RS-t	0.7336	0.9941	0.9116	0.9941
Ser	0.9987	0.8112	-	0.8112
Thr-t	0.7937	0.7941	-	0.8378
TIA	2.1930	2.1590	0.8602	2.1590
Tyr	1.8100	1.2140	0.9424	1.2140
Val	1.5410	1.0200	0.9219	1.0020
Cabrela and Córdoba				
	Kinship [a]	Eigenanalysis [b]	Kinship per chr [c]	Null [d]
Fat	1.5090	1.4990	0.82614	2.3170

CH—carbohydrates; RS—resistant starch; Ala—alanine; Arg—arginine; Asp—aspartic acid; Glu—glutamic acid; Gly—glycine; His—histidine; Ile—isoleucine; Leu—leucine; Lys—lysine; Met—methionine; Phe—phenylalanine; Pro—proline; Ser—serine; Thr—threonine; Tyr—tyrosine; Val—valine; TIA—trypsin inhibitor activity. [a] Model accounting for familial relatedness (K) [Phenotype = SNP + genotype + Error], with genotype random effects structured following a kinship matrix K. [b] Model accounting for population structure (Q) [Phenotype = Q + SNP + Error], using 15 principal components from the principal component analysis (PCA). [c] Model accounting for familial relatedness (K) [Phenotype = SNP + genotype + Error], using a different kinship matrix calculated for each chromosome using only the SNPs located on the remaining 10 chromosomes. [d] Model [Phenotype = SNP + Error], which does not account for any population structure or familial relatedness.

Table S6. SNP associations with 24 nutritional composition and protein quality traits under two contrasting environments (Cabrela and Córdoba). Original *p*-values (−log10 (*p*-value) ≥ 3) and the adjusted Benjamini-Yekutieli (BY) *p*-values are shown. Each separate table represents a different trait with data measured in a different environment (or both), each table contains an identification for the model used to account for genetic relatedness, marker positions within chromosomes (chr), allelic reference (more frequent) and allelic variant for the associated SNP, minor allele frequency (freq.), the effect of the allelic variant (rare allele), and the proportion of phenotypic variance explained by each associated SNP (Var_{QTL}/Var_{pheno}) detected using a panel of 106 Portuguese common bean accessions.

Trait: Fat

Environment: Cabrela and Córdoba

GWAS Model Using a Kinship Matrix Per Chromosome to Control for Population Structure

SNP Marker	Chr (Pv)	Position (bp)	−log10(*p*)	Original *p*-value	Adjusted BY *p*-value	Reference Allele	Rare Allele	Freq.	Effect	Std. Error	Var Explained by the SNP
SNP02929	7	4327459	5.207	6.21E−06	5.63E−05	C	A	0.293	0.163	0.038	0.167
DART05702	6	20043692	4.415	3.85E−05	1.13E−04	G	A	0.466	−0.120	0.030	0.109
DART02462	3	1864480	4.290	5.13E−05	1.69E−04	C	A	0.217	−0.246	0.063	0.313
DART05687	6	19671045	4.134	7.34E−05	2.25E−04	A	C	0.486	−0.125	0.033	0.119
DART05685	6	19656664	4.134	7.34E−05	2.81E−04	T	C	0.486	−0.125	0.033	0.119
DART05683	6	19650563	4.134	7.34E−05	3.38E−04	A	T	0.486	−0.125	0.033	0.119
DART05694	6	19859422	4.094	8.05E−05	3.94E−04	A	C	0.500	−0.116	0.030	0.103
DART05717	6	20282117	3.997	1.01E−04	4.50E−04	C	T	0.217	−0.116	0.031	0.070
DART04485	5	2514051	3.854	1.40E−04	5.07E−04	A	G	0.331	−0.134	0.036	0.121
DART05726	6	20358955	3.775	1.68E−04	5.63E−04	C	A	0.473	−0.112	0.031	0.096
DART10489	11	7612018	3.694	2.02E−04	6.19E−04	G	A	0.114	−0.128	0.035	0.050
SNP02689	6	18632623	3.624	2.38E−04	6.76E−04	A	G	0.500	−0.123	0.034	0.116
DART05684	6	19656562	3.359	4.37E−04	7.32E−04	T	C	0.486	−0.113	0.033	0.097
DART10592	11	12550185	3.359	4.38E−04	7.88E−04	T	G	0.408	−0.115	0.034	0.098
DART05716	6	20279035	3.264	5.45E−04	8.44E−04	A	C	0.283	−0.096	0.028	0.057
DART05729	6	20369688	3.262	5.47E−04	9.01E−04	C	T	0.426	−0.102	0.030	0.078
DART05727	6	20359051	3.262	5.47E−04	9.57E−04	G	A	0.437	−0.102	0.030	0.078

DART05730	6	20378740	3.255	5.56E−04	1.01E−03	G	T	0.227	−0.105	0.031	0.059
DART05737	6	20421345	3.197	6.36E−04	1.07E−03	C	T	0.442	−0.108	0.032	0.088
SNP01886	4	40645820	3.190	6.45E−04	1.13E−03	C	T	0.306	0.100	0.030	0.064
DART05651	6	18882026	3.099	7.96E−04	1.18E−03	A	T	0.407	0.092	0.028	0.062

Trait: Ash
Environment: Cabrela
GWAS Model Using a Kinship Matrix Per Chromosome to Control for Population Structure

SNP marker	Chro (Pv)	Position (bp)	−log10(*p*)	Original *p*-value	Adjusted BY *p*-value	Reference Allele	Rare Allele	Freq.	Effect	Std. Error	Var explained by the SNP
SNP01413	3	49082853	3.953	1.12E−04	5.63E−05	C	T	0.177	−0.084	0.023	0.297
SNP01983	5	167540	3.444	3.59E−04	1.13E−04	T	G	0.226	−0.068	0.020	0.238
SNP00192	1	24051047	3.265	5.43E−04	1.69E−04	A	C	0.479	0.040	0.012	0.114
DART03311	3	49016130	3.229	5.90E−04	2.25E−04	G	C	0.184	−0.075	0.023	0.245
SNP01408	3	48218792	3.084	8.24E−04	2.81E−04	G	A	0.190	−0.065	0.020	0.187
SNP01409	3	48659805	3.084	8.24E−04	3.38E−04	C	T	0.190	−0.065	0.020	0.187
SNP01412	3	48956711	3.084	8.24E−04	3.94E−04	A	G	0.190	−0.065	0.020	0.187
SNP00224	1	20329843	3.058	8.75E−04	4.50E−04	G	A	0.490	0.038	0.012	0.107
SNP00225	1	20443481	3.058	8.75E−04	5.07E−04	T	G	0.490	0.038	0.012	0.107
SNP00253	1	22620029	3.058	8.75E−04	5.63E−04	C	A	0.483	0.038	0.012	0.107
SNP00255	1	23387912	3.058	8.75E−04	6.19E−04	C	T	0.483	0.038	0.012	0.107
SNP00194	1	27915505	3.058	8.75E−04	6.76E−04	T	G	0.483	0.038	0.012	0.107
DART00388	1	20439837	3.051	8.89E−04	7.32E−04	T	G	0.320	−0.038	0.012	0.092

Trait: Fibre
Environment: Cabrela
GWAS Model Using a Kinship Matrix Per Chromosome to Control for Population Structure

SNP marker	Chr (Pv)	Position (bp)	−log10(*p*)	Original *p*-value	Adjusted BY *p*-value	Reference Allele	Rare Allele	Freq.	Effect	Std. Error	Var explained by the SNP
SNP03997	9	12018524	3.151	7.06E−04	5.63E-05	T	C	0.287	0.208	0.073	0.087

Trait: Carbohydrates-t
Environment: Cabrela
GWAS Model Using a Kinship Matrix Per Chromosome to Control for Population Structure

SNP marker	Chr (Pv)	Position (bp)	−log10(*p*)	Original *p*-value	Adjusted BY *p*-value	Reference Allele	Rare Allele	Freq.	Effect	Std. Error	Var explained by the SNP
SNP03273	7	37074930	4.514	3.06E−05	5.63E−05	A	C	0.331	−191364	48155	0.143
SNP04050	9	16552877	4.161	6.90E−05	1.13E−04	C	T	0.293	169478	44450	0.105
DART06856	7	32573518	4.109	7.77E−05	1.69E−04	T	C	0.275	−195797	51684	0.135
SNP03216	7	32982429	3.614	2.43E−04	2.25E−04	A	G	0.299	−177218	50021	0.117
SNP04726	10	41662474	3.605	2.48E−04	2.81E−04	T	G	0.497	156664	44276	0.109
DART03724	4	5477721	3.567	2.71E−04	3.38E−04	C	T	0.151	−291435	82830	0.192
DART00622	1	41657664	3.189	6.48E−04	3.94E−04	C	T	0.295	214522	64745	0.169
DART00521	1	36114290	3.164	6.85E−04	4.50E−04	A	T	0.234	166000	50311	0.088
DART06845	7	32033770	3.038	9.16E−04	5.07E−04	A	G	0.138	−186795	57879	0.073
SNP04178	9	29621879	3.027	9.40E−04	5.63E−04	G	A	0.286	−210755	65436	0.160
SNP04179	9	29659905	3.027	9.40E−04	6.19E−04	A	G	0.286	−210755	65436	0.160
SNP04180	9	29701960	3.027	9.40E−04	6.76E−04	A	G	0.286	−210755	65436	0.160
DART11240	11	51729967	3.023	9.49E−04	7.32E−04	A	T	0.234	−274861	85406	0.240

**Trait: Moisture**											
Environment: Cabrela											
GWAS Model Using a Kinship Matrix Per Chromosome to Control for Population Structure											
SNP marker	Chr (Pv)	Position (bp)	−log10(*p*)	Original *p*-value	Adjusted BY *p*-value	Reference Allele	Rare Allele	Freq.	Effect	Std. Error	Var explained by the SNP
**DART03318**	3	49214255	6.995	1.01E−07	5.63E−05	T	G	0.190	0.653	0.133	0.596
**SNP01413**	3	49082853	6.731	1.86E−07	1.13E−04	C	T	0.177	0.561	0.116	0.415
**DART03311**	3	49016130	6.438	3.65E−07	1.69E−04	C	G	0.184	0.546	0.116	0.405
**DART06920**	7	34647213	6.263	5.46E−07	2.25E−04	T	A	0.193	0.707	0.152	0.706
**DART03310**	3	49007246	5.852	1.41E−06	2.81E−04	T	C	0.173	0.445	0.099	0.257
**DART03271**	3	47685916	5.403	3.96E−06	3.38E−04	T	C	0.201	0.654	0.151	0.623
**SNP01343**	3	36254350	4.942	1.14E−05	3.94E−04	C	T	0.204	0.622	0.150	0.570
**DART03001**	3	36357086	4.834	1.46E−05	4.50E−04	T	G	0.211	0.622	0.151	0.584
**DART03010**	3	36757173	4.834	1.46E−05	5.07E−04	A	C	0.211	0.622	0.151	0.584
**DART03011**	3	36780366	4.834	1.46E−05	5.63E−04	C	T	0.211	0.622	0.151	0.584
**DART03021**	3	37085805	4.834	1.46E−05	6.19E−04	C	T	0.211	0.622	0.151	0.584
**SNP05275**	11	50975260	4.698	2.00E−05	6.76E−04	T	C	0.200	0.629	0.155	0.574
**DART11148**	11	50416350	4.698	2.00E−05	7.32E−04	G	A	0.191	0.629	0.155	0.555
**SNP01408**	3	48218792	4.307	4.93E−05	7.88E−04	G	A	0.190	0.398	0.102	0.221
**SNP01409**	3	48659805	4.307	4.93E−05	8.44E−04	C	T	0.190	0.398	0.102	0.221
**SNP01412**	3	48956711	4.307	4.93E−05	9.01E−04	A	G	0.190	0.398	0.102	0.221
**DART03285**	3	47970262	4.162	6.89E−05	9.57E−04	G	T	0.199	0.390	0.102	0.220
**DART03281**	3	47944825	4.162	6.89E−05	1.01E−03	A	G	0.197	0.390	0.102	0.218
**DART03295**	3	48346869	4.162	6.89E−05	1.07E−03	C	G	0.197	0.390	0.102	0.218
**DART03298**	3	48482771	4.162	6.89E−05	1.13E−03	T	A	0.197	0.390	0.102	0.218
**DART03300**	3	48561346	4.162	6.89E−05	1.18E−03	G	C	0.197	0.390	0.102	0.218
**DART03301**	3	48568167	4.162	6.89E−05	1.24E−03	C	A	0.197	0.390	0.102	0.218
**DART03302**	3	48625130	4.162	6.89E−05	1.29E−03	G	A	0.197	0.390	0.102	0.218
**DART03304**	3	48728557	4.162	6.89E−05	1.35E−03	T	C	0.197	0.390	0.102	0.218
**DART03305**	3	48777063	4.162	6.89E−05	1.41E−03	C	T	0.197	0.390	0.102	0.218
**DART03305**	3	48777063	4.162	6.89E−05	1.46E−03	C	T	0.197	0.390	0.102	0.218
**DART03012**	3	36780357	4.111	7.75E−05	1.52E−03	T	A	0.230	0.521	0.138	0.435
**DART03287**	3	48114098	4.090	8.13E−05	1.58E−03	T	A	0.178	0.384	0.101	0.195
**DART03313**	3	49151179	3.912	1.22E−04	1.63E−03	G	T	0.456	−0.200	0.058	0.090
**DART03314**	3	49151215	3.912	1.22E−04	1.69E−03	G	T	0.206	0.343	0.100	0.174
**DART03316**	3	49159646	3.912	1.22E−04	1.75E−03	C	G	0.490	−0.220	0.064	0.109
**DART03724**	4	5477721	3.845	1.43E−04	1.80E−03	C	T	0.218	0.409	0.125	0.258
**DART03315**	3	49154661	3.671	2.13E−04	1.86E−03	T	A	0.490	−0.220	0.064	0.109
**DART06929**	7	34810203	3.593	2.55E−04	1.91E−03	C	A	0.219	0.405	0.124	0.255
**SNP00192**	1	24051047	3.504	3.13E−04	1.97E−03	A	C	0.116	0.426	0.115	0.168
**DART06927**	7	34800654	3.411	3.88E−04	2.03E−03	C	A	0.490	−0.209	0.064	0.099
**DART06928**	7	34800747	3.411	3.88E−04	2.08E−03	A	T	0.143	0.304	0.094	0.103
**DART04935**	5	36009330	3.409	3.90E−04	2.14E−03	C	G	0.218	0.409	0.125	0.258
**DART03299**	3	48561306	3.406	3.92E−04	2.20E−03	G	A	0.483	−0.220	0.064	0.109
**SNP00224**	1	20329843	3.392	4.05E−04	2.25E−03	G	A	0.116	0.426	0.115	0.168
**SNP00225**	1	20443481	3.392	4.05E−04	2.31E−03	T	G	0.116	0.426	0.115	0.168
**SNP00253**	1	22620029	3.392	4.05E−04	2.36E−03	C	A	0.151	0.418	0.114	0.202
**SNP00255**	1	23387912	3.392	4.05E−04	2.42E−03	C	T	0.119	0.412	0.115	0.161
**SNP00194**	1	27915505	3.392	4.05E−04	2.48E−03	T	G	0.308	0.264	0.075	0.135
**DART06925**	7	34778584	3.372	4.24E−04	2.53E−03	A	T	0.449	−0.184	0.057	0.076

**SNP01402**	3	46701407	3.176	6.67E−04	2.59E−03	C	T	0.483	−0.220	0.064	0.109
**SNP01404**	3	47181179	3.176	6.67E−04	2.65E−03	C	T	0.483	−0.220	0.064	0.109
**SNP01407**	3	47713882	3.176	6.67E−04	2.70E−03	A	G	0.122	0.257	0.075	0.064
**SNP00189**	1	15602573	3.134	7.34E−04	2.76E−03	C	T	0.479	−0.222	0.064	0.111
**SNP00208**	1	17567333	3.134	7.34E−04	2.81E−03	G	A	0.313	0.253	0.074	0.125
**DART03248**	3	46473960	3.106	7.84E−04	2.87E−03	A	T	0.211	0.410	0.124	0.253
**DART03255**	3	46561309	3.106	7.84E−04	2.93E−03	T	C	0.211	0.410	0.124	0.253
**DART03259**	3	46833288	3.106	7.84E−04	2.98E−03	A	T	0.211	0.410	0.124	0.253
**DART03260**	3	46835346	3.106	7.84E−04	3.04E−03	T	G	0.497	0.209	0.064	0.099
**DART03267**	3	47235277	3.106	7.84E−04	3.10E−03	C	G	0.497	0.209	0.064	0.099
**DART03274**	3	47731395	3.106	7.84E−04	3.15E−03	G	A	0.226	0.409	0.125	0.265
**DART03275**	3	47766981	3.106	7.84E−04	3.21E−03	A	G	0.226	0.409	0.125	0.265
**DART03279**	3	47838454	3.106	7.84E−04	3.27E−03	T	C	0.218	0.409	0.125	0.258
**DART03265**	3	47017893	3.093	8.07E−04	3.32E−03	A	G	0.218	0.409	0.125	0.258
**DART00373**	1	18693507	3.085	8.23E−04	3.38E−03	G	A	0.313	0.253	0.074	0.125
**DART03303**	3	48626311	3.021	9.53E−04	3.43E−03	T	C	0.218	0.409	0.125	0.258
**SNP02354**	5	33184977	3.017	9.61E−04	3.49E−03	C	T	0.218	0.409	0.125	0.258

Trait: Protein
Environment: Cabrela
GWAS Model Using a Kinship Matrix Per Chromosome to Control for Population Structure

SNP marker	Chr (Pv)	Position (bp)	−log10(p)	Original p-value	Adjusted BY p-value	Reference Allele	Rare Allele	Freq.	Effect	Std. Error	Var explained by the SNP
SNP03273	7	37074930	4.209	6.18E−05	5.63E−05	A	C	0.335	0.664	0.173	0.095
SNP04726	10	41662474	4.189	6.47E−05	1.13E−04	T	G	0.491	−0.624	0.163	0.094
DART06856	7	32573518	4.150	7.08E−05	1.69E−04	T	C	0.287	0.699	0.183	0.097
SNP03216	7	32982429	3.939	1.15E−04	2.25E−04	A	G	0.281	0.748	0.202	0.109
SNP04050	9	16552877	3.839	1.45E−04	2.81E−04	C	T	0.293	−0.693	0.190	0.096
DART06845	7	32033770	3.802	1.58E−04	3.38E−04	A	G	0.138	0.827	0.227	0.078
DART06714	7	23085248	3.050	8.91E−04	3.94E−04	T	A	0.275	0.580	0.179	0.065

Trait: Resistant Starch
Environment: Cabrela
GWAS Model Using a Kinship Matrix Per Chromosome to Control for Population Structure

SNP marker	Chr (Pv)	Position (bp)	−log10(p)	Original p-value	Adjusted BY p-value	Reference Allele	Rare Allele	Freq.	Effect	Std. Error	Var explained by the SNP
DART00521	1	36114290	4.552	2.81E−05	5.63E−05	A	T	0.219	2.155	0.601	0.130
DART03644	4	3196591	3.779	1.66E−04	1.13E−04	T	A	0.219	−4.024	1.111	0.454
DART03649	4	3277042	3.779	1.66E−04	1.69E−04	T	C	0.218	−4.024	1.111	0.452
DART03651	4	3326628	3.779	1.66E−04	2.25E−04	C	T	0.286	−2.109	0.587	0.149
DART03653	4	3454402	3.779	1.66E−04	2.81E−04	A	T	0.212	−3.653	1.034	0.366
SNP01084	3	534165	3.722	1.90E−04	3.38E−04	A	G	0.221	−4.024	1.111	0.456
DART03647	4	3233976	3.601	2.50E−04	3.94E−04	C	A	0.226	−3.451	0.993	0.342
DART03654	4	3509199	3.493	3.21E−04	4.50E−04	T	G	0.320	−1.577	0.491	0.089
SNP00254	1	23507640	3.018	9.59E−04	5.07E−04	G	A	0.218	−4.024	1.111	0.452

Trait: Arginine-t
Environment: Córdoba
GWAS Model Using a Kinship Matrix Per Chromosome to Control for Population Structure

SNP marker	Chr (Pv)	Position (bp)	−log10(*p*)	Original *p*-value	Adjusted BY *p*-value	Reference Allele	Rare Allele	Freq.	Effect	Std. Error	Var explained by the SNP
SNP00732	2	24255711	3.926	1.19E−04	5.63E−05	G	A	0.265	0.046	0.013	0.203
SNP00621	2	10579918	3.724	1.89E−04	1.13E−04	G	A	0.327	0.032	0.009	0.110
SNP00622	2	10839301	3.724	1.89E−04	1.69E−04	G	A	0.320	0.032	0.009	0.109
SNP00631	2	11786780	3.684	2.07E−04	2.25E−04	G	A	0.374	0.032	0.009	0.116
SNP00859	2	33656184	3.504	3.13E−04	2.81E−04	C	T	0.177	0.053	0.015	0.196
SNP00726	2	23273420	3.365	4.31E−04	3.38E−04	C	A	0.272	0.045	0.013	0.192
SNP00727	2	23497729	3.365	4.31E−04	3.94E−04	G	T	0.272	0.045	0.013	0.192
SNP00728	2	23568315	3.365	4.31E−04	4.50E−04	C	T	0.272	0.045	0.013	0.192
SNP00735	2	24349083	3.365	4.31E−04	5.07E−04	C	A	0.272	0.045	0.013	0.192
SNP00737	2	24498317	3.365	4.31E−04	5.63E−04	A	G	0.272	0.045	0.013	0.192
SNP00602	2	7726390	3.356	4.40E−04	6.19E−04	G	T	0.401	0.030	0.009	0.103
SNP00722	2	23003775	3.301	5.00E−04	6.76E−04	C	A	0.247	0.040	0.012	0.144
SNP00723	2	23018208	3.301	5.00E−04	7.32E−04	C	T	0.247	0.040	0.012	0.144
SNP00725	2	23106531	3.301	5.00E−04	7.88E−04	A	C	0.247	0.040	0.012	0.144
SNP00674	2	16723568	3.271	5.36E−04	8.44E−04	A	G	0.389	0.031	0.009	0.109
SNP00632	2	11809539	3.246	5.68E−04	9.01E−04	C	A	0.381	0.031	0.009	0.107
SNP00636	2	12320956	3.246	5.68E−04	9.57E−04	A	G	0.381	0.031	0.009	0.107
SNP00654	2	14074599	3.246	5.68E−04	1.01E−03	A	G	0.381	0.031	0.009	0.107
SNP00655	2	14160962	3.246	5.68E−04	1.07E−03	C	T	0.381	0.031	0.009	0.107
SNP00657	2	14279732	3.246	5.68E−04	1.13E−03	A	G	0.381	0.031	0.009	0.107
SNP00658	2	14385480	3.246	5.68E−04	1.18E−03	A	C	0.381	0.031	0.009	0.107
SNP00663	2	15636955	3.246	5.68E−04	1.24E−03	G	A	0.381	0.031	0.009	0.107
SNP00664	2	15678936	3.246	5.68E−04	1.29E−03	G	A	0.381	0.031	0.009	0.107
SNP00676	2	16900143	3.246	5.68E−04	1.35E−03	A	G	0.381	0.031	0.009	0.107
SNP00680	2	16969001	3.246	5.68E−04	1.41E−03	A	G	0.381	0.031	0.009	0.107
SNP00695	2	18021030	3.246	5.68E−04	1.46E−03	T	C	0.381	0.031	0.009	0.107
SNP00698	2	18879988	3.246	5.68E−04	1.52E−03	G	A	0.381	0.031	0.009	0.107
SNP00699	2	20578461	3.246	5.68E−04	1.58E−03	C	T	0.381	0.031	0.009	0.107
SNP00641	2	12676791	3.239	5.77E−04	1.63E−03	A	G	0.422	0.029	0.009	0.102
SNP00757	2	25498132	3.173	6.71E−04	1.69E−03	T	C	0.231	0.052	0.016	0.236
SNP00785	2	26688811	3.173	6.71E−04	1.75E−03	A	G	0.238	0.052	0.016	0.241
SNP00787	2	26761844	3.173	6.71E−04	1.80E−03	G	A	0.238	0.052	0.016	0.241

Trait: Alanine-t

Environment: Córdoba

GWAS Model Using a Kinship Matrix Per Chromosome to Control for Population Structure

SNP marker	Chr (Pv)	Position (bp)	−log10(*p*)	Original *p*-value	Adjusted BY *p*-value	Reference Allele	Rare Allele	Freq.	Effect	Std. Error	Var explained by the SNP
SNP04308	9	37988166	4.828	1.49E−05	5.63E−05	C	T	0.106	0.029	0.007	0.125
DART09201	9	37991684	4.435	3.67E−05	1.13E−04	A	G	0.112	0.026	0.007	0.107

Trait: Ash

Environment: Córdoba

GWAS Model Using a Kinship Matrix Per Chromosome to Control for Population Structure

SNP marker	Chr (Pv)	Position (bp)	−log10(*p*)	Original *p*-value	Adjusted BY *p*-value	Reference Allele	Rare Allele	Freq.	Effect	Std. Error	Var explained by the SNP
SNP00484	1	50635589	3.104	7.87E−04	5.63E−05	C	T	0.340	−0.060	0.018	0.091

SNP03984	9	11168802	3.032	9.28E−04	1.13E−04	G	A	0.211	−0.058	0.018	0.063

Trait: Aspartic acid

Environment: Córdoba

GWAS Model Using a Kinship Matrix Per Chromosome to Control for Population Structure

SNP marker	Chr (Pv)	Position (bp)	−log10(p)	Original p-value	Adjusted BY p-value	Reference Allele	Rare Allele	Freq.	Effect	Std. Error	Var explained by the SNP
SNP00747	2	25240802	3.469	3.40E−04	5.63E−05	G	T	0.111	0.916	0.265	0.107
DART01478	2	24738549	3.438	3.65E−04	1.13E−04	C	A	0.106	0.802	0.233	0.079
SNP00739	2	24785706	3.438	3.65E−04	1.69E−04	G	A	0.106	0.802	0.233	0.079
SNP00741	2	24878778	3.438	3.65E−04	2.25E−04	A	G	0.106	0.802	0.233	0.079
DART01499	2	25383556	3.199	6.32E−04	2.81E−04	C	T	0.109	0.936	0.282	0.110

Trait: Carbohydrates-t

Environment: Córdoba

GWAS Model Using a Kinship Matrix Per Chromosome to Control for Population Structure

SNP marker	Chr (Pv)	Position (bp)	−log10(p)	Original p-value	Adjusted BY p-value	Reference Allele	Rare Allele	Freq.	Effect	Std. Error	Var explained by the SNP
SNP01507	4	2151313	4.482	3.29E−05	5.63E−05	T	G	0.136	218993	54895	0.117
SNP01495	4	1610074	4.307	4.93E−05	1.13E−04	T	C	0.130	212584	54397	0.106
SNP01505	4	2116992	4.307	4.93E−05	1.69E−04	A	G	0.130	212584	54397	0.106
DART03583	4	1618009	3.973	1.06E−04	2.25E−04	T	G	0.103	214936	57367	0.088
DART03569	4	1389584	3.958	1.10E−04	2.81E−04	T	C	0.137	213870	57202	0.112
SNP01490	4	1389673	3.958	1.10E−04	3.38E−04	C	T	0.136	213870	57202	0.111
SNP01497	4	1738973	3.820	1.51E−04	3.94E−04	A	G	0.136	197615	53850	0.095
SNP00248	1	25794601	3.100	7.94E−04	4.50E−04	T	C	0.367	−131503	40122	0.083

Trait: Fibre

Environment: Córdoba

GWAS Model Using a Kinship Matrix Per Chromosome to Control for Population Structure

SNP marker	Chr (Pv)	Position (bp)	−log10(p)	Original p-value	Adjusted BY p-value	Reference Allele	Rare Allele	Freq.	Effect	Std. Error	Var explained by the SNP
DART01343	2	13149249	3.386	4.11E−04	5.63E−05	T	C	0.301	0.293	0.085	0.069

Trait: Glutamic Acid

Environment: Córdoba

GWAS Model Using a Kinship Matrix Per Chromosome to Control for Population Structure

No significant Associations

Trait: Glycine

Environment: Córdoba

GWAS Model Using a Kinship Matrix Per Chromosome to Control for Population Structure

No significant associations

**Trait: Histidine-t**
Environment: Córdoba
GWAS Model Using 15 Principal Components to Control for Population Structure
**No Significant Associations**

**Trait: Isoleucine-t**
Environment: Córdoba
GWAS Model Using a Kinship Matrix Per Chromosome to Control for Population Structure
**No Significant Associations**

**Trait: Leucine-t**
Environment: Córdoba
GWAS Model Using a Kinship Matrix Per Chromosome to Control for Population Structure
**No Significant Associations**

Trait: Lysine

Environment: Córdoba

GWAS Model Using 15 Principal Components to Control for Population Structure

**SNP marker**	**Chr (Pv)**	**Position (bp)**	**−log10(*p*)**	***p*-value**	**Adjusted BY *p*-value**	**Reference allele**	**Rare allele**	**Freq.**	**Effect**	**Std. Error**	**Var explained by the SNP**
SNP04308	9	37988166	3.825	1.50E−04	5.63E−05	C	T	0.109	0.506	0.139	0.102
DART09201	9	37991684	3.035	9.23E−04	1.13E−04	A	G	0.116	0.422	0.131	0.075

**Trait: Methionine**
Environment: Córdoba
GWAS Model Using a Kinship Matrix Per Chromosome to Control for Population Structure
**No Significant Associations**

Trait: Moisture

Environment: Córdoba

GWAS Model Using a Kinship Matrix Per Chromosome to Control for Population Structure

**SNP marker**	**Chr (Pv)**	**Position (bp)**	**−log10(*p*)**	**Original *p*-value**	**Adjusted BY *p*-value**	**Reference Allele**	**Rare Allele**	**Freq.**	**Effect**	**Std. Error**	**Var explained by the SNP**
DART03724	4	5477721	3.658	2.20E−04	5.63E−05	C	T	0.151	−0.303	0.084	0.090
SNP03990	9	11586533	3.417	3.83E−04	1.13E−04	A	G	0.299	0.188	0.055	0.057
SNP03991	9	11592150	3.417	3.83E−04	1.69E−04	C	A	0.299	0.188	0.055	0.057
SNP03441	9	11805398	3.210	6.16E−04	2.25E−04	C	T	0.279	0.184	0.055	0.052
DART10454	11	6506568	3.209	6.18E−04	2.81E−04	C	T	0.156	−0.259	0.078	0.068
SNP02381	5	37516499	3.209	6.19E−04	3.38E−04	A	C	0.240	−0.188	0.056	0.049

Trait: Phenylalanine

Environment: Córdoba

GWAS Model Using 15 Principal Components to Control for Population Structure

**SNP marker**	**Chr (Pv)**	**Position (bp)**	**−log10(*p*)**	**Original *p*-value**	**Adjusted BY *p*-value**	**Reference Allele**	**Rare Allele**	**Freq.**	**Effect**	**Std. Error**	**Var explained by the SNP**

DART01478	2	24738549	3.731	1.86E−04	5.63E−05	C	A	0.106	0.355	0.108	0.069
SNP00739	2	24785706	3.731	1.86E−04	1.13E−04	G	A	0.106	0.355	0.108	0.069
SNP00741	2	24878778	3.731	1.86E−04	1.69E−04	A	G	0.106	0.355	0.108	0.069
SNP04308	9	37988166	3.342	4.55E−04	2.25E−04	C	T	0.106	0.339	0.109	0.063

Trait: Protein-t

Environment: Córdoba

GWAS Model Using a Kinship Matrix Per Chromosome to Control for Population Structure

**SNP marker**	**Chr (Pv)**	**Position (bp)**	**−log10(*p*)**	**Original *p*-value**	**Adjusted BY *p*-value**	**Reference Allele**	**Rare Allele**	**Freq.**	**Effect**	**Std. Error**	**Var explained by the SNP**
DART01929	2	39912206	3.846	1.42E−04	5.63E−05	C	G	0.418	0.0003	0.0001	0.152
SNP01507	4	2151313	3.834	1.47E−04	1.13E−04	T	G	0.136	−0.0004	0.0001	0.107
DART03569	4	1389584	3.585	2.60E−04	1.69E−04	T	C	0.137	−0.0004	0.0001	0.106
SNP01490	4	1389673	3.585	2.60E−04	2.25E−04	C	T	0.136	−0.0004	0.0001	0.106
SNP01495	4	1610074	3.542	2.87E−04	2.81E−04	T	C	0.130	−0.0004	0.0001	0.091
SNP01505	4	2116992	3.542	2.87E−04	3.38E−04	A	G	0.130	−0.0004	0.0001	0.091
DART11136	11	50289095	3.542	2.87E−04	3.94E−04	C	A	0.295	−0.0003	0.0001	0.084
DART03583	4	1618009	3.414	3.86E−04	4.50E−04	T	G	0.103	−0.0004	0.0001	0.079
DART01951	2	40819431	3.405	3.93E−04	5.07E−04	T	C	0.421	0.0003	0.0001	0.128
SNP01497	4	1738973	3.305	4.95E−04	5.63E−04	A	G	0.136	−0.0003	0.0001	0.086
SNP05238	11	48731137	3.113	7.71E−04	6.19E−04	G	T	0.500	0.0002	0.0001	0.083

Trait: Proline

Environment: Córdoba

GWAS Model Using a Kinship Matrix Per Chromosome to Control for Population Structure

No Significant Associations

Trait: Resistant Starch-t

Environment: Córdoba

GWAS Model Using a Kinship Matrix Per Chromosome to Control for Population Structure

**SNP marker**	**Chr (Pv)**	**Position (bp)**	**−log10(*p*)**	**Original *p*-value**	**Adjusted BY *p*-value**	**Reference Allele**	**Rare Allele**	**Freq.**	**Effect**	**Std. Error**	**Var explained by the SNP**
SNP01672	4	7305468	4.434	3.68E−05	5.63E−05	C	T	0.435	−0.275	0.069	0.086
DART03741	4	7783059	4.319	4.80E−05	1.13E−04	T	C	0.396	−0.285	0.073	0.091
SNP01686	4	9007160	4.102	7.91E−05	1.69E−04	G	A	0.450	0.269	0.071	0.083
SNP01678	4	8026040	4.056	8.79E−05	2.25E−04	T	C	0.380	−0.263	0.069	0.076
DART03743	4	8057141	4.009	9.79E−05	2.81E−04	A	G	0.382	−0.263	0.070	0.076
DART03764	4	8661499	3.847	1.42E−04	3.38E−04	A	T	0.493	−0.260	0.070	0.078
DART03809	4	10324736	3.406	3.93E−04	3.94E−04	A	G	0.455	0.264	0.077	0.081
SNP01694	4	10284225	3.355	4.42E−04	4.50E−04	T	C	0.497	0.241	0.070	0.068
SNP01698	4	10617156	3.355	4.42E−04	5.07E−04	T	C	0.497	0.241	0.070	0.068

Trait: Serine

Environment: Córdoba

GWAS Model Using 15 Principal Components to Control for Population Structure

**SNP marker**	**Chr (Pv)**	**Position (bp)**	**−log10(*p*)**	**Original *p*-value**	**Adjusted BY *p*-value**	**Reference Allele**	**Rare Allele**	**Freq.**	**Effect**	**Std. Error**	**Var explained by the SNP**

DART09201	9	37991684	3.204	6.25E−04	5.63E−05	A	G	0.112	0.498	0.162	0.080

Trait: Threonine-t

Environment: Córdoba

GWAS Model Using 15 Principal Components to Control for Population Structure

No Significant Associations

Trait: Trypsin inhibitor activity

Environment: Córdoba

GWAS Model Using a Kinship Matrix Per Chromosome to Control for Population Structure

SNP marker	Chr (Pv)	Position (bp)	−log10(*p*)	Original *p*-value	Adjusted BY *p*-value	Reference allele	Rare allele	Freq.	Effect	Std. Error	Var explained by the SNP
SNP01601	4	4044489	3.997	1.01E−04	5.63E−05	G	A	0.422	−1.322	0.355	0.122
SNP03386	8	6272513	3.776	1.68E−04	1.13E−04	T	C	0.197	−2.008	0.555	0.182
SNP00304	1	3.7E+07	3.322	4.77E−04	1.69E−04	A	G	0.429	−1.210	0.358	0.102
DART07380	8	5534904	3.056	8.79E−04	2.25E−04	A	G	0.454	1.141	0.353	0.092
SNP02678	6	1.7E+07	3.026	9.42E−04	2.81E−04	T	C	0.158	−1.337	0.416	0.068

Trait: Tyrosine

Environment: Córdoba

GWAS Model Using a Kinship Matrix Per Chromosome to Control for Population Structure

SNP marker	Chr (Pv)	Position (bp)	−log10(*p*)	Original *p*-value	Adjusted BY *p*-value	Reference allele	Rare allele	Freq.	Effect	Std. Error	Var explained by the SNP
DART01478	2	24738549	3.498	3.18E−04	5.63E−05	C	A	0.103	0.300	0.086	0.083
SNP00739	2	24785706	3.498	3.18E−04	1.13E−04	G	A	0.103	0.300	0.086	0.083
SNP00741	2	24878778	3.498	3.18E−04	1.69E−04	A	G	0.103	0.300	0.086	0.083
SNP00797	2	26991724	3.325	4.73E−04	2.25E−04	T	G	0.137	0.312	0.092	0.115
SNP00802	2	27104809	3.325	4.73E−04	2.81E−04	A	C	0.137	0.312	0.092	0.115
SNP00747	2	25240802	3.282	5.22E−04	3.38E−04	G	T	0.109	0.328	0.098	0.104
DART01499	2	25383556	3.282	5.22E−04	3.94E−04	T	C	0.109	0.328	0.098	0.104

Trait: Valine

Environment: Córdoba

GWAS Model Using a Kinship Matrix Per Chromosome to Control for Population Structure

SNP marker	Chr (Pv)	Position (bp)	−log10(*p*)	Original *p*-value	Adjusted BY *p*-value	Reference allele	Rare allele	Freq.	Effect	Std. Error	Var explained by the SNP
SNP04308	9	37988166	5.293	5.09E−06	5.63E−05	C	T	0.106	0.460	0.107	0.138
DART09201	9	37991684	4.375	4.22E−05	1.13E−04	A	G	0.112	0.395	0.101	0.106
DART02434	3	1337996	3.143	7.19E−04	1.69E−04	C	T	0.345	−0.211	0.064	0.069

Table S7. Putative candidate genes for the 16 nutritional composition and protein quality traits for the significant (−log10 (*p*-value) ≥ 3) SNP-trait associations under two contrasting environments (Cabrela and Córdoba). Each separate table includes a different trait with data measured in a different environment (or both), and the model used to account for genetic relatedness. Putative candidate genes based on the gene annotation for the *P. vulgaris* genome v2.1, respective protein annotation (KEG, PANTHER or Pfam), and MapMan pathway or functional characterization are indicated.

Trait: Fat

Environment: Cabrela and Córdoba

GWAS Model Using a Kinship Matrix Per Chromosome to Control for Population Structure

SNP Marker	Associated Gene	Gene Location	Protein Annotation	Pathway

DART02462	Phvul.003G019300	Chr03:1860289.1864791 forward	methyltransferases	RNA processing
SNP01886	no candidate gene			
DART04485	no candidate gene			
DART05651	Phvul.006G076700 (gene model within LD block)	Chr06:18874264.18875459 reverse	allergen-related	Phytohormone action
DART05716	Phvul.006G091200	Chr06:20275192.20280509 reverse	zinc finger (Ran-binding) family protein	RNA processing
DART05729	Phvul.006G091900	Chr06:20369564.20371924 reverse	Chaperone DnaJ-domain superfamily protein	Not assigned
DART05727	Phvul.006G091700	Chr06:20357709.20364237 reverse	Metallopeptidase M24 family protein	Enzyme classification
DART05730	Phvul.006G091922	Chr06:20377746.20378995 reverse	Tetratricopeptide repeat (TPR)-like superfamily protein	Not assigned
DART05737	Phvul.006G092700	Chr06:20421328.20423088 reverse	Glycoprotein membrane precursor GPI-anchored	Not assigned
DART05726	Phvul.006G091700	Chr06:20357709.20364237 reverse	Metallopeptidase M24 family protein	Enzyme classification
DART05684	Phvul.006G085100	Chr06:19653016.19657410 forward	ARM repeat superfamily protein	Not assigned
DART05694	Phvul.006G086600	Chr06:19854770.19859573 forward	Ankyrin repeat family protein	Not assigned
DART05717	Phvul.006G091300	Chr06:20281869.20304890 reverse	recovery protein 3	Not assigned
DART05702	Phvul.006G088600	Chr06:20043205.20044667 forward	alpha/beta-Hydrolases superfamily protein	Not assigned
SNP02689	Phvul.006G073600	Chr06:18630394.18633885 forward	cation/H+ exchanger 24	Solute transport
DART05687	Phvul.006G085300	Chr06:19664847.19671971 reverse	P-loop containing nucleoside triphosphate hydrolases superfamily protein	Not assigned
DART05685	Phvul.006G085100	Chr06:19653016.19657410 forward	ARM repeat superfamily protein	Not assigned
DART05683	Phvul.006G085000	Chr06:19648148.19652450 reverse	Class I glutamine amidotransferase-like superfamily protein	Not assigned
SNP02929	no candidate gene			
DART10592	Phvul.011G107200	Chr11:12546726.12551394 reverse	terpene synthase-like sequence-1,8-cineole	Secondary metabolism
DART10489	Phvul.011G081700	Chr11:7603587.7613411 reverse	ATP-dependent helicase family protein	Not assigned

Trait: Ash

Environment: Cabrela

GWAS Model Using a Kinship Matrix Per Chromosome to Control for Population Structure

SNP marker	Associated Gene	Gene Location	Protein Annotation	Pathway
SNP00192	no candidate gene			
SNP00224	Phvul.001G100500 (gene model within LD block)	Chr01:20437455.20440118 forward	Arabidopsis NAC domain containing protein 87	RNA biosynthesis
SNP00225	Phvul.001G100500 (gene model within LD block)	Chr01:20437455.20440118 forward	Arabidopsis NAC domain containing protein 87	RNA biosynthesis
SNP00253	no candidate gene			

SNP00255	no candidate gene			
SNP00194	no candidate gene			
DART00388	Phvul.001G100500	Chr01:20437455.20440118 forward	Arabidopsis NAC domain containing protein 87	RNA biosynthesis
SNP01413	Phvul.003G251800	Chr03:49081487.49083269 forward	homeobox protein 40	RNA biosynthesis
DART03311	no candidate gene			
SNP01408	Phvul.003G245800 (gene model within LD block)	Chr03:48262801.48279790 reverse	Transducin/WD40 repeat-like superfamily protein	Vesicle trafficking
SNP01409	Phvul.003G249100	Chr03:48658456.48701569 forward	Protein of unknown function (DUF1162)	Not assigned
SNP01412	no candidate gene			
SNP01983	Phvul.005G002100	Chr05:162586.168574 forward	translocase inner membrane subunit 44-2	Not assigned

Trait: Fibre

Environment: Cabrela

GWAS Model Using a Kinship Matrix Per Chromosome to Control for Population Structure

SNP marker	Associated Gene	Gene Location	Protein Annotation	Pathway
SNP03997	Phvul.005G015100	Chr05:1277028.1281680 forward	cysteine-rich RLK (RECEPTOR-like protein kinase) 10	Enzyme classification

Trait: Carbohydrates-t

Environment: Cabrela

GWAS Model Using a Kinship Matrix Per Chromosome to Control for Population Structure

SNP marker	Associated Gene	Gene Location	Protein Annotation	Pathway
DART00521	no candidate gene			
DART00622	Phvul.001G162000	Chr01:41656979.41666085 reverse	ribophorin II (RPN2) family protein	Protein modification
DART03724	Phvul.004G045900	Chr04:5475003.5478151 forward	galacturonosyltransferase 9	Not assigned
DART06845	Phvul.007G197000	Chr07:32032979.32034534 forward	rapid alkalinization factor 1	Phytohormone action
DART06856	Phvul.007G202700	Chr07:32563434.32580790 forward	Metallopeptidase M24 family protein	Protein modification
SNP03216	Phvul.007G207200	Chr07:32978086.32983259 forward	DEAD box RNA helicase (RH3)	Not assigned
SNP03273	Phvul.007G248100 (gene model within LD block)	Chr07:37098956.37107864 reverse	S-adenosyl-L-methionine-dependent methyltransferases superfamily protein	Not assigned
SNP04050	Phvul.009G106600	Chr09:16550489.16553821 forward	La protein 1	Not assigned
SNP04178	no candidate gene			
SNP04179	no candidate gene			
SNP04180	no candidate gene			
SNP04726	Phvul.010G134400	Chr10:41660542.41662815 forward	hydroxyproline-rich glycoprotein family protein	Not assigned
DART11240	Phvul.011G201600	Chr11:51724617.51730352 forward	No annotation	Not assigned

SNP marker	Associated Gene	Gene Location	Protein Annotation	Pathway
**Trait: Moisture**				
Environment: Cabrela				
GWAS Model Using a Kinship Matrix Per Chromosome to Control for Population Structure				
SNP00192	no candidate gene			
SNP00224	Phvul.001G100500 (gene model within LD block)	Chr01:20437455.20440118 forward	Arabidopsis NAC domain containing protein 87	RNA biosynthesis
SNP00225	Phvul.001G100500 (gene model within LD block)	Chr01:20437455.20440118 forward	Arabidopsis NAC domain containing protein 87	RNA biosynthesis
SNP00253	no candidate gene			
SNP00255	no candidate gene			
SNP00194	no candidate gene			
SNP00189	no candidate gene			
SNP00208	Phvul.001G094400	Chr01:17566408.17568080 forward	Galactose oxidase/kelch repeat superfamily protein	Not assigned
DART00373	Phvul.001G097600	Chr01:18690176.18718445 forward	ARM repeat superfamily protein	Not assigned
DART03271	no candidate gene			
DART03318	no candidate gene			
DART03001	no candidate gene			
DART03010	no candidate gene			
DART03011	Phvul.003G152900	Chr03:36779067.36784453 reverse	Leucine-rich repeat protein kinase family protein	Enzyme classification
DART03021	Phvul.003G155200	Chr03:37082724.37086061 forward	zinc ion binding;nucleic acid binding	Not assigned
SNP01343	no candidate gene			
DART03012	Phvul.003G152900	Chr03:36779067.36784453 reverse	Leucine-rich repeat protein kinase family protein	Enzyme classification
SNP01413	Phvul.003G251800	Chr03:49081488.49083269 forward	homeobox protein 40	RNA biossynthesis
DART03311	no candidate gene			
DART03310	no candidate gene			
SNP01408	Phvul.003G245800 (gene model within LD block)	Chr03:48262932.48279685 reverse	Transducin/WD40 repeat-like superfamily protein	Vesicle trafficking
SNP01409	Phvul.003G249100	Chr03:48658457.48701569 forward	Protein of unknown function (DUF1162)	Not assigned
SNP01412	no candidate gene			
DART03285	no candidate gene			
DART03281	Phvul.003G243500	Chr03:47940038.47944953 forward	alpha/beta-Hydrolases superfamily protein	Lipid metabolism
DART03295	Phvul.003G246600	Chr03:48345587.48349491 reverse	Ribosomal protein L25/Gln-tRNA synthetase, anti-codon-binding domain	Not assigned
DART03298	Phvul.003G247700	Chr03:48482464.48486019 forward	SKU5 similar 17	Enzyme classification
DART03300	Phvul.003G248200	Chr03:48561200.48564505 forward	methyl esterase 1	Not assigned
DART03301	Phvul.003G248300	Chr03:48567955.48569848 forward	methyl esterase 1	Not assigned
DART03302	Phvul.003G248800	Chr03:48621346.48626836 reverse	Prolyl oligopeptidase family protein	Not assigned
DART03304	Phvul.003G249600	Chr03:48726640.48729993 forward	Phototropic-responsive NPH3 family protein	Not assigned

DART03305	Phvul.003G249600 (gene model within LD block)	Chr03:48726640.48729993 forward	Phototropic-responsive NPH3 family protein	Not assigned
DART03287	no candidate gene			
DART03305	Phvul.003G250400 (gene model within LD block)	Chr03:48855134.48859964 forward	No annotation	Not assigned
DART03313	Phvul.003G252300	Chr03:49149038.49158015 reverse	Protein phosphatase 2C family protein	Not assigned
DART03314	Phvul.003G252300	Chr03:49149038.49158015 reverse	Protein phosphatase 2C family protein	Not assigned
DART03316	Phvul.003G252300 (gene model within LD block)	Chr03:49149038.49158015 reverse	Protein phosphatase 2C family protein	Not assigned
DART03315	Phvul.003G252300	Chr03:49149038.49158015 reverse	Protein phosphatase 2C family protein	Not assigned
DART03299	Phvul.003G248200	Chr03:48561199.48564505 forward	methyl esterase 1	Not assigned
SNP01402	no candidate gene			
SNP01404	no candidate gene			
SNP01407	Phvul.003G242100	Chr03:47713460.47720866 forward	Plant protein of unknown function (DUF869)	Not assigned
DART03248	no candidate gene			
DART03255	no candidate gene			
DART03259	Phvul.003G234900	Chr03:46832570.46838216 forward	Arabinanase/levansucrase/invertase	Cell wall organisation
DART03260	Phvul.003G234900	Chr03:46832570.46838216 forward	Arabinanase/levansucrase/invertase	Cell wall organisation
DART03267	no candidate gene			
DART03274	Phvul.003G242300	Chr03:47729681.47735528 forward	PLC-like phosphodiesterases superfamily protein	Not assigned
DART03275	Phvul.003G242500	hr03:47764488.47768173 forward	no annotation	Not assigned
DART03279	Phvul.003G243000	Chr03:47832773.47842353 forward	nicotinate phosphoribosyltransferase 2	Not assigned
DART03265	Phvul.003G236300	Chr03:47016837.47018758 forward	GRAS family transcription factor	RNA biosynthesis
DART03303	Phvul.003G248800	Chr03:48621345.48626836 reverse	Prolyl oligopeptidase family protein	Not assigned
DART03724	Phvul.004G045900	Chr04:5475002.5478151 forward	galacturonosyltransferase 9	Not assigned
DART04935	Phvul.005G122800	Chr05:36006617.36009521 forward	no annotation	
SNP02354	no candidate gene			
DART06920	Phvul.007G223101	Chr07:34646520.34647668 forward	PHYTOSULFOKINE 3 PRECURSOR	Phytohormone action
DART06929	Phvul.007G224400 (gene model within LD block)	Chr07:34800272.34805489 reverse	aspartate/glutamate/uridylate kinase family protein	RNA biosynthesis
DART06927	Phvul.007G224400	Chr07:34800272.34805489 reverse	aspartate/glutamate/uridylate kinase family protein	RNA biosynthesis
DART06928	Phvul.007G224400	Chr07:34800272.34805489 reverse	aspartate/glutamate/uridylate kinase family protein	RNA biosynthesis
DART06925	Phvul.007G224400	Chr07:34800272.34805489 reverse	aspartate/glutamate/uridylate kinase family protein	RNA biosynthesis
SNP05275	no candidate gene			

DART11148	Phvul.011G190400	Chr11:50411188.50420407 reverse	Leucine carboxyl methyltransferase	Not assigned

Trait: Protein

Environment: Cabrela

GWAS Model Using a Kinship Matrix Per Chromosome to Control for Population Structure

SNP marker	Associated Gene	Gene Location	Protein Annotation	Pathway
DART06845	Phvul.004G045900	Chr04:5475003.5478151 forward	galacturonosyltransferase 9	Not assigned
DART06856	no candidate gene			
SNP03216	Phvul.007G207200	Chr07:32978086.32983259 forward	DEAD box RNA helicase (RH3)	Not assigned
SNP03273	Phvul.009G111500	Chr09:17246244.17251134 forward	NADP-malic enzyme 3	Not assigned
DART06714	Phvul.007G142050	Chr07:23075517.23087032 reverse	haemoglobin 2	Not assigned
SNP04050	Phvul.009G106600	Chr09:16550488.16553821 forward	La protein 1	Not assigned
SNP04726	Phvul.010G134400	Chr10:41660542.41662815 forward	hydroxyproline-rich glycoprotein family protein	Not assigned

Trait: Resistant Starch

Environment: Cabrela

GWAS Model Using a Kinship Matrix Per Chromosome to Control for Population Structure

SNP marker	Associated Gene	Gene Location	Protein Annotation	Pathway
DART00521	no candidate gene			
SNP00254	no candidate gene			
SNP01084	no candidate gene			
DART03644	no candidate gene			
DART03649	Phvul.004G028400	Chr04:3276634.3279896 reverse	Protein of unknown function (DUF1666)	Not asssigned
DART03651	no candidate gene			
DART03653	Phvul.004G029000	Chr04:3442039.3454624 forward	structural maintenance of chromosome 3	Not asssigned
DART03647	Phvul.004G027900	Chr04:3229736.3234269 forward	cullin 3	Protein homeostasis
DART03654	Phvul.004G029600	Chr04:3507344.3510240 reverse	SPX domain gene 4	Nutrient uptake

Trait: Arginine-t

Environment: Córdoba

GWAS Model Using a Kinship Matrix Per Chromosome to Control for Population Structure

SNP marker	Associated Gene	Gene Location	Protein Annotation	Pathway
SNP00732	Phvul.002G113000	Chr02:24255078.24259120 reverse	Transmembrane amino acid transporter family protein	Not assigned
SNP00621	Phvul.002G072100 (gene model within LD block)	Chr02:10523962.10527817 reverse	aldehyde dehydrogenase 2B7	Not assigned
SNP00622	Phvul.002G074200 (gene model within LD block)	Chr02:11104765.11107990 forward	sterol 4-alpha-methyl-oxidase 2-1	Not assigned

SNP00631	no candidate gene			
SNP00859	no candidate gene			
SNP00726	Phvul.002G108800	Chr02:23272599.23274318 forward	Chalcone-flavanone isomerase family protein	Not assigned
SNP00727	Phvul.002G021900 (gene model within LD block)	Chr02:2324324.2327806 forward	SKU5 similar 17	Enzyme classification
SNP00728	no candidate gene			
SNP00735	Phvul.002G113000 (gene model within LD block)	Chr02:24255077.24259120 reverse	Transmembrane amino acid transporter family protein	Not assigned
SNP00737	Phvul.002G114400	Chr02:24493540.24499071 forward	acyl activating enzyme 1	Not assigned
SNP00602	Phvul.002G064300 (gene model within LD block)	Chr02:7727150.7732404 reverse	Plant protein of unknown function (DUF828)	Not assigned
SNP00722	Phvul.002G107700	Chr02:23002898.23004170 reverse	Rhodanese/Cell cycle control phosphatase superfamily protein	Not assigned
SNP00723	Phvul.002G107700 (gene model within LD block)	Chr02:23002898.23004170 reverse	Rhodanese/Cell cycle control phosphatase superfamily protein	Not assigned
SNP00725	Phvul.002G108700 (gene model within LD block)	Chr02:23210281.23216106 reverse	calcium-dependent protein kinase 16	Enzyme classification
SNP00674	no candidate gene			
SNP00632	Phvul.002G077700 (gene model within LD block)	Chr02:11945221.11953149 reverse	Rhodanese/Cell cycle control phosphatase superfamily protein	RNA processing
SNP00636	Phvul.002G079200 (gene model within LD block)	Chr02:12311751.12319211 forward	transmembrane receptors;ATP binding	External stimuli response
SNP00654	Phvul.002G086500 (gene model within LD block)	Chr02:14052424.14061593 forward	Mog1/PsbP/DUF1795-like photosystem II reaction center PsbP family protein	Not assigned
SNP00655	no candidate gene			
SNP00657	no candidate gene			
SNP00658	no candidate gene			
SNP00663	no candidate gene			
SNP00664	no candidate gene			
SNP00676	Phvul.002G092600 (gene model within LD block)	Chr02:16923877.16927223 forward	ARM repeat superfamily protein	Protein homeostasis
SNP00680	no candidate gene			
SNP00695	no candidate gene			
SNP00698	no candidate gene			
SNP00699	Phvul.002G100300 (gene model within LD block	Chr02:20807292.20811588 forward	Pentatricopeptide repeat (PPR) superfamily protein	RNA processing
SNP00641	no candidate gene			

SNP00757	no candidate gene			
SNP00785	no candidate gene			
SNP00787	Phvul.002G126600 (gene model within LD block	Chr02:26820121.26823012 reverse	Leucine-rich repeat protein kinase family protein	Enzyme classification

Trait: Alanine-t

Environment: Córdoba

GWAS Model Using a Kinship Matrix Per Chromosome to Control for Population Structure

SNP marker	Associated Gene	Gene location	Protein annotation	Pathway
SNP04308	Phvul.009G245800	Chr09:36694249.36700838 forward	Transcription factor GTE6	Not assigned
DART09201	no candidate gene			

Trait: Ash

Environment: Córdoba

GWAS Model Using a Kinship Matrix Per Chromosome to Control for Population Structure

SNP marker	Associated Gene	Gene Location	Protein Annotation	Pathway
SNP00484	Phvul.001G257500	Chr01:50634941.50641381 reverse	G-protein coupled receptors;GTPase activators	Multi-process regulation / External stimuli
SNP03984	Phvul.009G061400	Chr09:11167885.11171330 reverse	cytochrome P450, family 82, subfamily C, polypeptide 4	Enzyme classification

Trait: Aspartic acid

Environment: Córdoba

GWAS Model Using a Kinship Matrix Per Chromosome to Control for Population Structure

SNP marker	Associated Gene	Gene Location	Protein Annotation	Pathway
SNP00747	Phvul.002G118400	Chr02:25240760.25244027 reverse	plant intracellular ras group-related LRR 4	Not assigned
DART01478	Phvul.002G115600	Chr02:24736175.24747468 forward	Phosphoinositide phosphatase family protein	Not assigned
SNP00739	Phvul.002G115900	Chr02:24785118.24788258 reverse	Protein kinase superfamily protein	Enzyme classification
SNP00741	Phvul.002G116400 (gene model within LD block)	Chr02:24857037.24862543 reverse	Rab escort protein	Vesicle trafficking
DART01499	Phvul.002G118900	Chr02:25381775.25385993 forward	hydroxy methylglutaryl CoA reductase 1	Secondary metabolism

Trait: Carbohydrates-t

Environment: Córdoba

GWAS Model Using a Kinship Matrix Per Chromosome to Control for Population Structure

SNP marker	Associated Gene	Gene Location	Protein Annotation	Pathway
SNP00248	no candidate gene			
SNP01507	Phvul.004G017600 (gene model within LD block)	Chr04:2107717.2121994 reverse	methyl-CPG-binding domain 9	Not assigned

DART03583	Phvul.004G014600	Chr04:1616741.1619302 reverse	formate dehydrogenase	Enzyme classification
DART03569	Phvul.004G012600	Chr04:1385799.1398207 reverse	Protein kinase superfamily protein	Enzyme classification
SNP01490	Phvul.004G012600	Chr04:1385799.1398207 reverse	Protein kinase superfamily protein	Enzyme classification
SNP01495	Phvul.004G014500	Chr04:1609805.1616040 reverse	Prenyltransferase family protein	Not assigned
SNP01505	Phvul.004G017600	Chr04:2107717.2121994 reverse	methyl-CPG-binding domain 9	Not assigned
SNP01497	Phvul.004G016000	Chr04:1735410.1741124 reverse	NB-ARC domain-containing disease resistance protein	Not assigned

Trait: Fibre

Environment: Córdoba

GWAS Model Using a Kinship Matrix Per Chromosome to Control for Population Structure

SNP marker	Associated Gene	Gene Location	Protein Annotation	Pathway
DART01343	Phvul.002G083000	Chr02:13147125.13154633 reverse	Chaperone protein htpG family protein	Not assigned

Trait: Lysine

Environment: Córdoba

GWAS Model Using 15 Principal Components to Control for Population Structure

SNP marker	Associated Gene	Gene Location	Protein Annotation	Pathway
SNP04308	Phvul.009G245800	Chr09:36694249.36700838 forward	Transcription factor GTE6	Not assigned
DART09201	no candidate gene			

Trait: Moisture

Environment: Córdoba

GWAS Model Using a Kinship Matrix Per Chromosome to Control for Population Structure

SNP marker	Associated Gene	Gene Location	Protein Annotation	Pathway
DART03724	Phvul.004G045900	Chr04:5475003.5478151 forward	galacturonosyltransferase 9	Not assigned
SNP02381	Phvul.005G134600 (gene model within LD block)	Chr05:37493349.37503175 reverse	SIT4 phosphatase-associated family protein	Not assigned
SNP03990	no candidate gene			
SNP03991	no candidate gene			
SNP03441	no candidate gene			
DART10454	Phvul.011G071200	Chr11:6499214.6507438 reverse	RNA binding (RRM/RBD/RNP motifs) family protein	RNA processing

Trait: Phenylalanine

Environment: Córdoba

GWAS Model Using 15 Principal Components To Control For Population Structure

SNP marker	Associated Gene	Gene Location	Protein Annotation	Pathway
DART01478	Phvul.002G115600	Chr02:24736175.24747468 forward	Phosphoinositide phosphatase family protein	Not assigned

SNP00739	Phvul.002G115900	Chr02:24785118.24788258 reverse	Protein kinase superfamily protein	Enzyme classification
SNP00741	Phvul.002G116400 (gene model within LD block)	Chr02:24857037.24862543 reverse	Rab escort protein	Vesicle trafficking
SNP04308	no candidate gene			

Trait: Protein-t

Environment: Córdoba

GWAS Model Using a Kinship Matrix Per Chromosome to Control for Population Structure

SNP marker	Associated Gene	Gene Location	Protein Annotation	Pathway
DART01929	Phvul.002G227600	Chr02:39905710.39923744 forward	sensitive to freezing 6	RNA biosynthesis
DART01951	no candidate gene			
SNP01497	Phvul.004G016000	Chr04:1735410.1741124 reverse	NB-ARC domain-containing disease resistance protein	Not assigned
SNP01495	Phvul.004G014500	Chr04:1609805.1616040 reverse	Prenyltransferase family protein	Not assigned
SNP01505	Phvul.004G017600	Chr04:2107717.2121994 reverse	methyl-CPG-binding domain 9	Not assigned
DART03583	Phvul.004G014600	Chr04:1616741.1619302 reverse	formate dehydrogenase	Enzyme classification
DART03569	Phvul.004G012600	Chr04:1385799.1398207 reverse	Protein kinase superfamily protein	Enzyme classification
SNP01490	Phvul.004G012600	Chr04:1385799.1398207 reverse	Protein kinase superfamily protein	Enzyme classification
SNP01507	Phvul.004G017600	Chr04:2107717.2121994 reverse	methyl-CPG-binding domain 9	Not assigned
SNP05238	Phvul.011G176500	Chr11:48728040.48733070 forward	Transducin/WD40 repeat-like superfamily protein	Not assigned
**DART11136**	Phvul.011G189400	Chr11:50287046.50292073 reverse	No annotation	Not assigned

Trait: Resistant Starch-t

Environment: Córdoba

GWAS Model Using a Kinship Matrix Per Chromosome to Control for Population Structure

SNP marker	Associated Gene	Gene Location	Protein Annotation	Pathway
SNP01672	no candidate gene			
DART03741	Phvul.004G056800 (gene model within LD block)	Chr04:7780496.7784354 reverse	Ankyrin repeat family protein	Not assigned
SNP01678	Phvul.004G058000	Chr04:8051898.8060708 forward	No annotation	Cell wall organization
DART03743	no candidate gene			
SNP01686	Phvul.004G056800	Chr04:7780496.7784354 reverse	Ankyrin repeat family protein	Not assigned
DART03764	Phvul.004G062100	Chr04:8659209.8665946 reverse	putative recombination initiation defect 1	Cell division

DART03809	Phvul.004G068100	Chr04:10321676.10330707 forward	Protein of unknown function (DUF803)	Not assigned
SNP01694	Phvul.004G068100 (gene model within LD block)	Chr04:10321676.10330707 forward	Protein of unknown function (DUF803)	Not assigned
SNP01698	no candidate gene			

Trait: Serine

Environment: Córdoba

GWAS Model Using 15 Principal Components to Control for Population Structure

SNP marker	Associated Gene	Gene Location	Protein Annotation	Pathway
DART09201	no candidate gene			

Trait: Trypsin inhibitor activity

Environment: Córdoba

GWAS Model Using a Kinship Matrix Per Chromosome to Control for Population Structure

SNP marker	Associated Gene	Gene Location	Protein Annotation	Pathway
SNP00304	Phvul.001G132400	Chr01:36687478.36688902 forward	No annotation	Not assigned
SNP01601	Phvul.004G034300	Chr04:3999142.4003023 reverse	biotin carboxyl carrier protein 2	Lipid metabolism
SNP02678	Phvul.006G061900	Chr06:17061726.17067400 reverse	Transducin/WD40 repeat-like superfamily protein	Not assigned
DART07380	Phvul.008G061000	Chr08:5525568.5537937 reverse	transcription regulatory protein SNF2, putative	Not assigned
SNP03386	no candidate gene			

Trait: Tyrosine

Environment: Córdoba

GWAS Model Using a Kinship Matrix Per Chromosome to Control for Population Structure

SNP marker	Associated Gene	Gene location	Protein annotation	Pathway
DART01478	Phvul.002G115600	Chr02:24736175.24747468 forward	Phosphoinositide phosphatase family protein	Not assigned
SNP00739	Phvul.002G115900	Chr02:24785118.24788258 reverse	Protein kinase superfamily protein	Enzyme classification
SNP00741	Phvul.002G116400 (gene model within LD block)	Chr02:24857037.24862543 reverse	Rab escort protein	Vesicle trafficking
SNP00747	Phvul.002G118400	Chr02:25240760.25244027 reverse	plant intracellular ras group-related LRR 4	Not assigned
DART01499	Phvul.002G118900	Chr02:25381776.25385993 forward	hydroxy methylglutaryl CoA reductase 1	Secondary metabolism
SNP00797	no candidate gene			
SNP00802	Phvul.002G128700	Chr02:27104699.27109270 reverse	no annotation	Not assigned

Trait: Valine

Environment: Córdoba

GWAS Model Using a Kinship Matrix Per Chromosome to Control for Population Structure				
**SNP marker**	**Associated Gene**	**Gene Location**	**Protein Annotation**	**Pathway**
DART02434	Phvul.003G013100	Chr03:1336511.1347083 reverse	tobamovirus multiplication protein 3	Not assigned
SNP04308	Phvul.009G245800	Chr09:36694249.36700838 forward	Transcription factor GTE6	Not assigned
DART09201	no candidate gene			

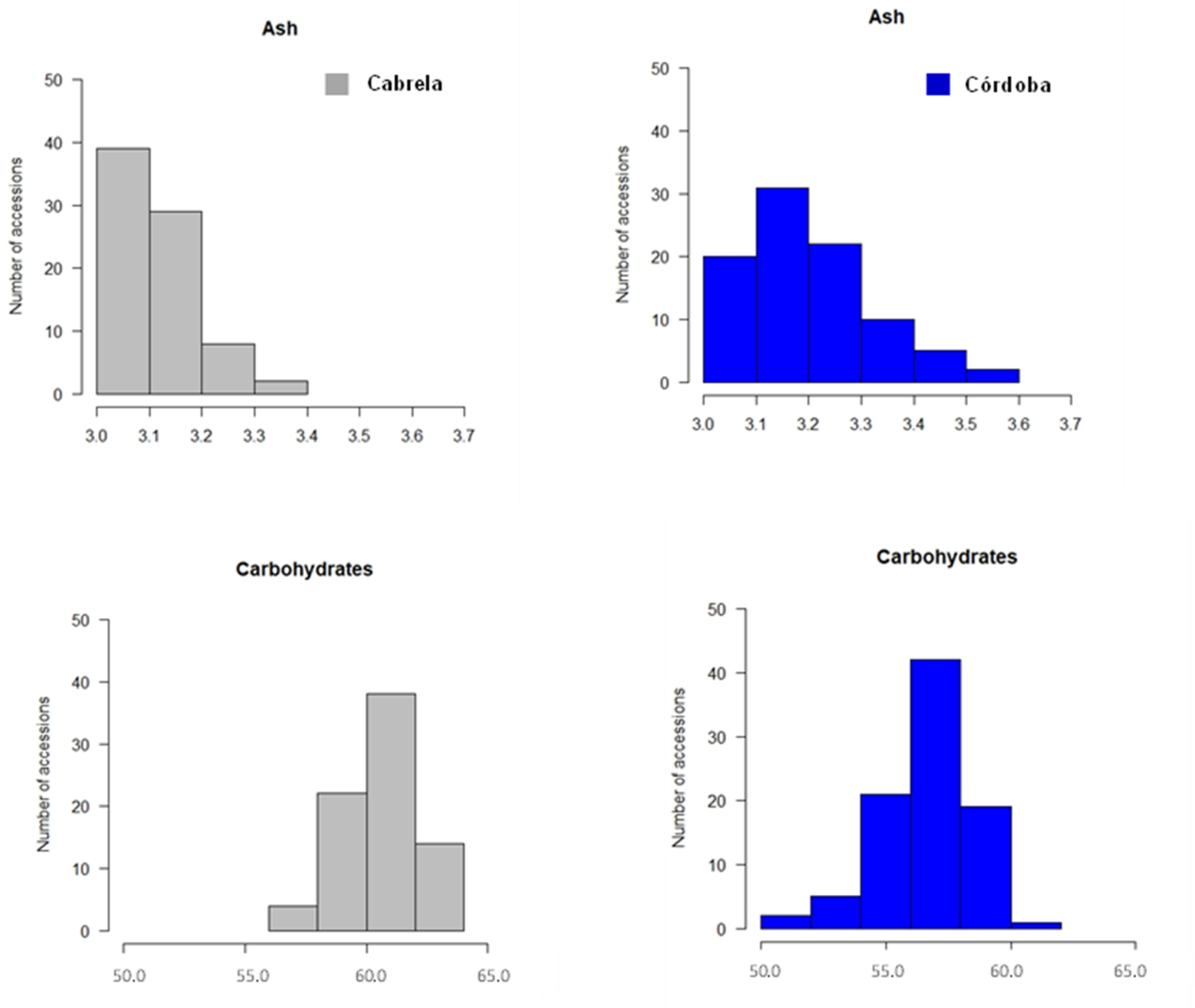

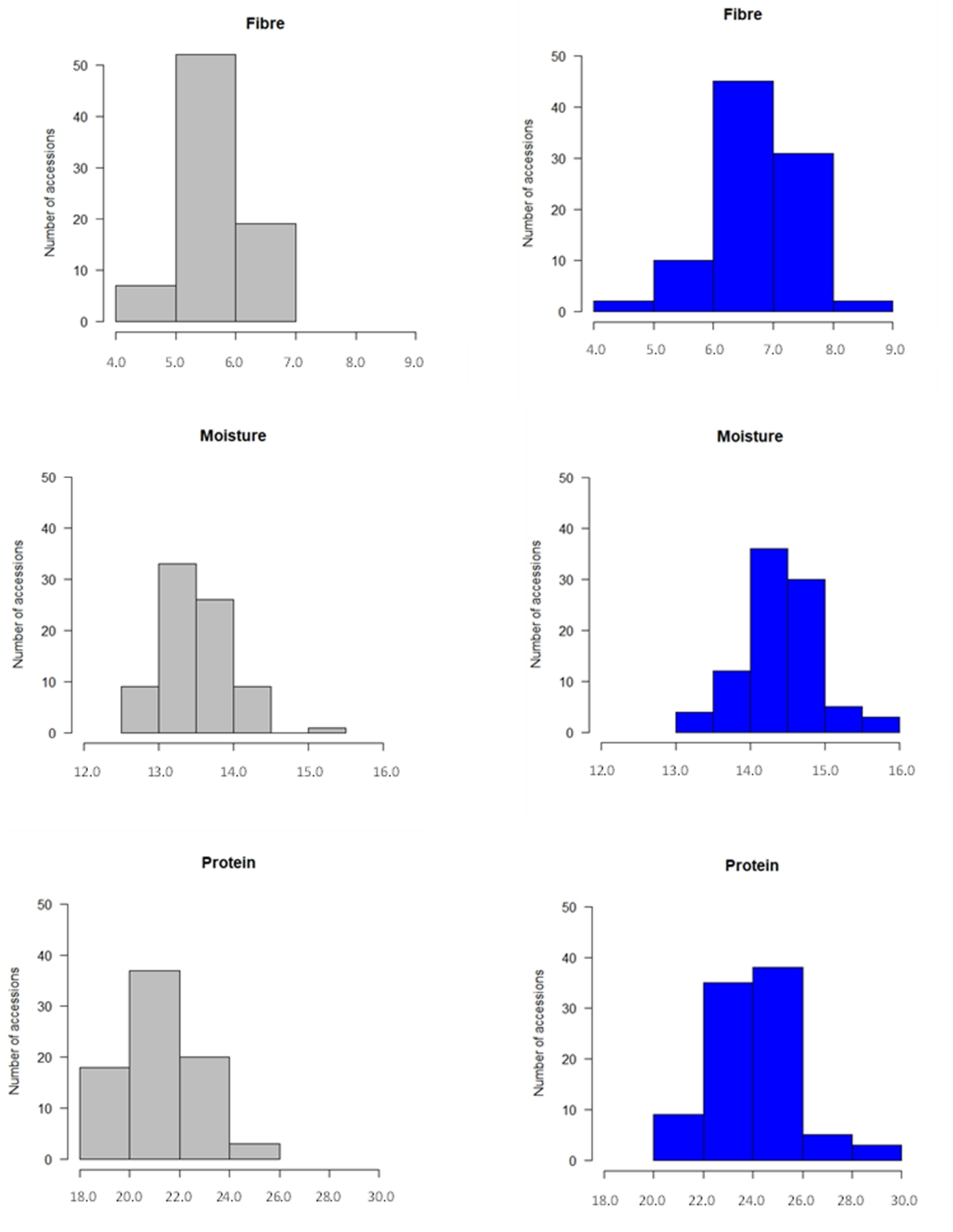
Fibre
Number of accessions
0
10
20
30
40
50
4.0
5.0
6.0
7.0
8.0
9.0
Fibre
Number of accessions
Moisture
Number of accessions
12.0
13.0
14.0
15.0
16.0
Moisture
Number of accessions
Protein
Number of accessions
18.0
20.0
22.0
24.0
26.0
28.0
30.0
Protein
Number of accessions

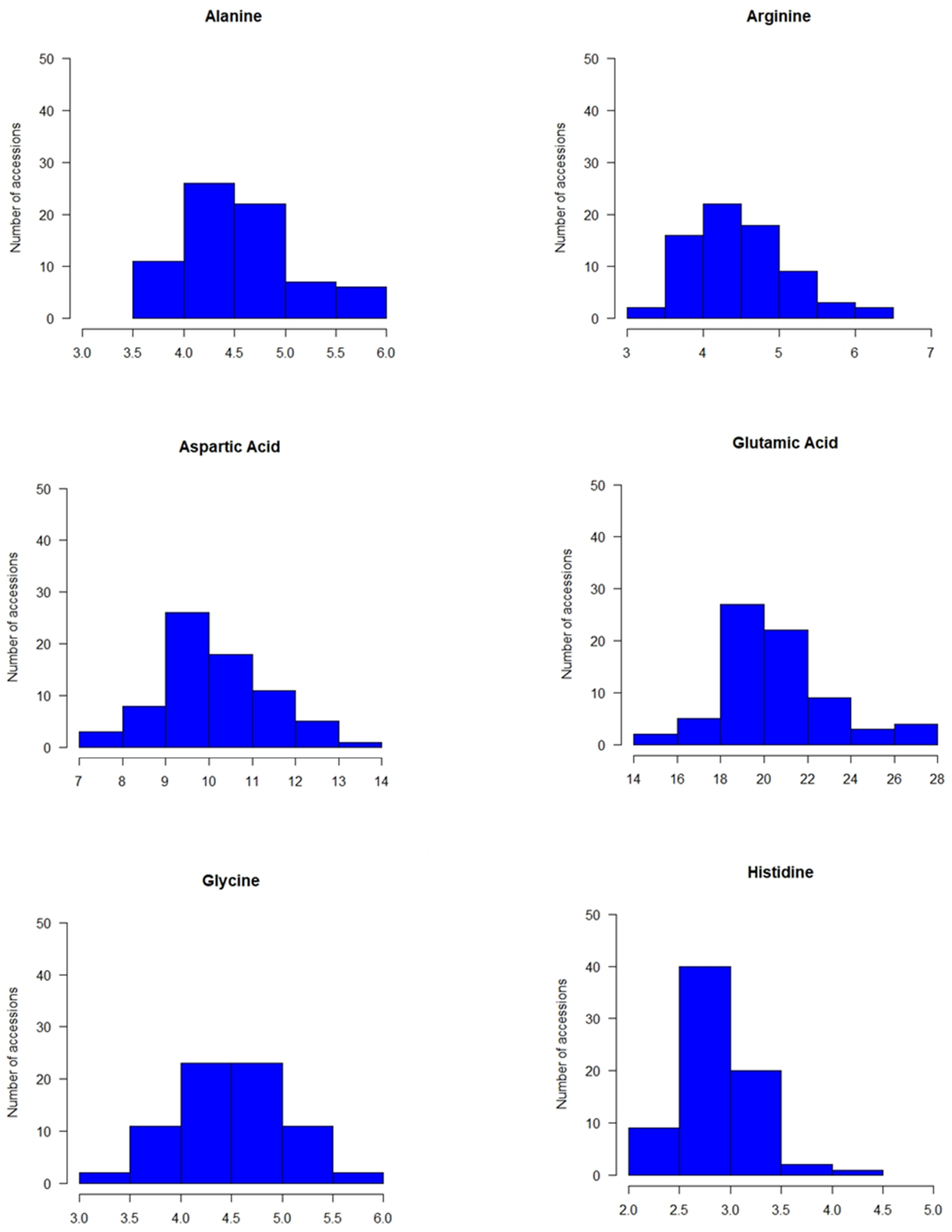
Alanine
Number of accessions
0 10 20 30 40 50
3.0 3.5 4.0 4.5 5.0 5.5 6.0
Arginine
Number of accessions
0 10 20 30 40 50
3 4 5 6 7
Aspartic Acid
Number of accessions
0 10 20 30 40 50
7 8 9 10 11 12 13 14
Glutamic Acid
Number of accessions
0 10 20 30 40 50
14 16 18 20 22 24 26 28
Glycine
Number of accessions
0 10 20 30 40 50
3.0 3.5 4.0 4.5 5.0 5.5 6.0
Histidine
Number of accessions
0 10 20 30 40 50
2.0 2.5 3.0 3.5 4.0 4.5 5.0

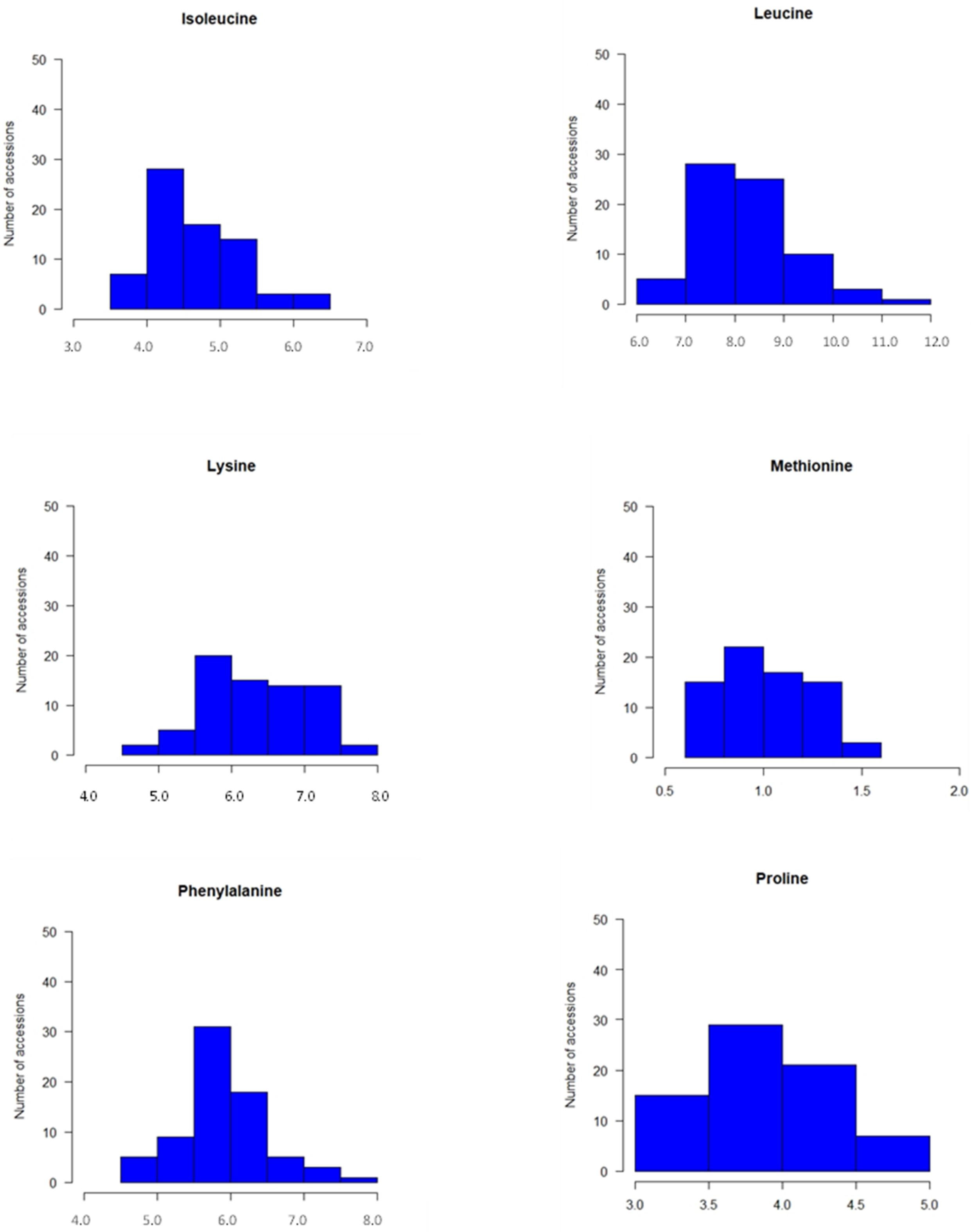
Isoleucine
Leucine
Lysine
Methionine
Phenylalanine
Proline
Number of accessions
0
10
20
30
40
50
3.0
4.0
5.0
6.0
7.0
6.0
7.0
8.0
9.0
10.0
11.0
12.0
4.0
5.0
6.0
7.0
8.0
0.5
1.0
1.5
2.0
4.0
5.0
6.0
7.0
8.0
3.0
3.5
4.0
4.5
5.0

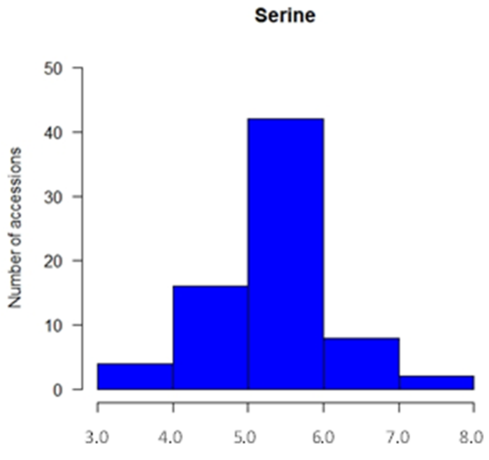
Serine
Number of accessions
0
10
20
30
40
50
3.0
4.0
5.0
6.0
7.0
8.0

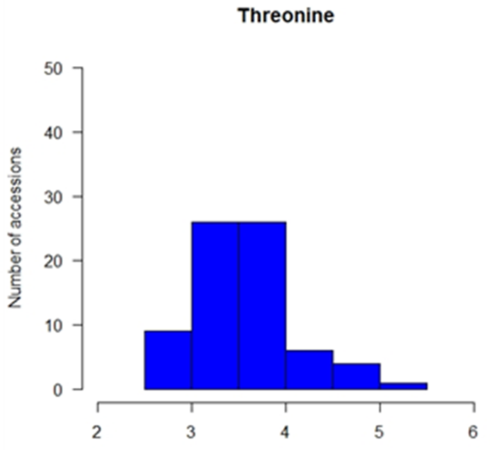
Threonine
Number of accessions
0
10
20
30
40
50
2
3
4
5
6

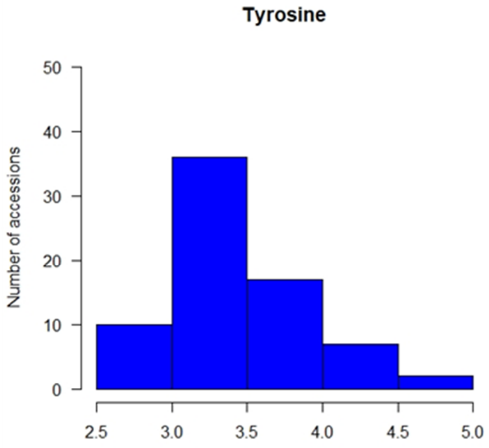
Tyrosine
Number of accessions
0
10
20
30
40
50
2.5
3.0
3.5
4.0
4.5
5.0

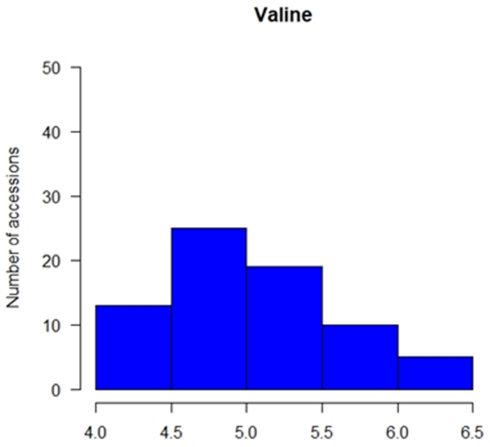
Valine
Number of accessions
0
10
20
30
40
50
4.0
4.5
5.0
5.5
6.0
6.5

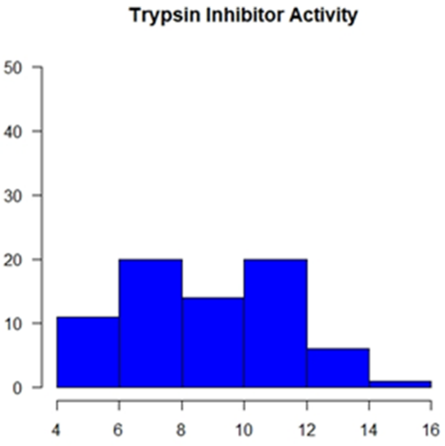
Trypsin Inhibitor Activity
0
10
20
30
40
50
4
6
8
10
12
14
16

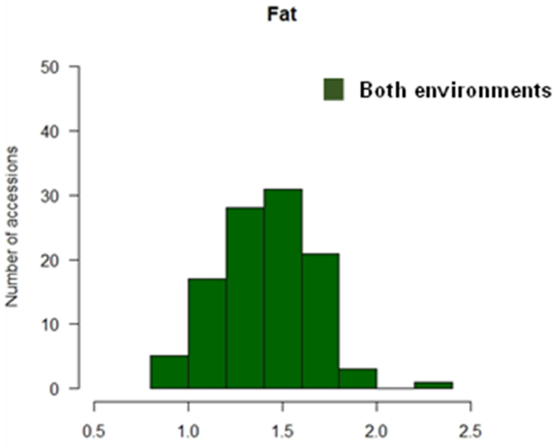
Fat
Both environments
Number of accessions
0
10
20
30
40
50
0.5
1.0
1.5
2.0
2.5

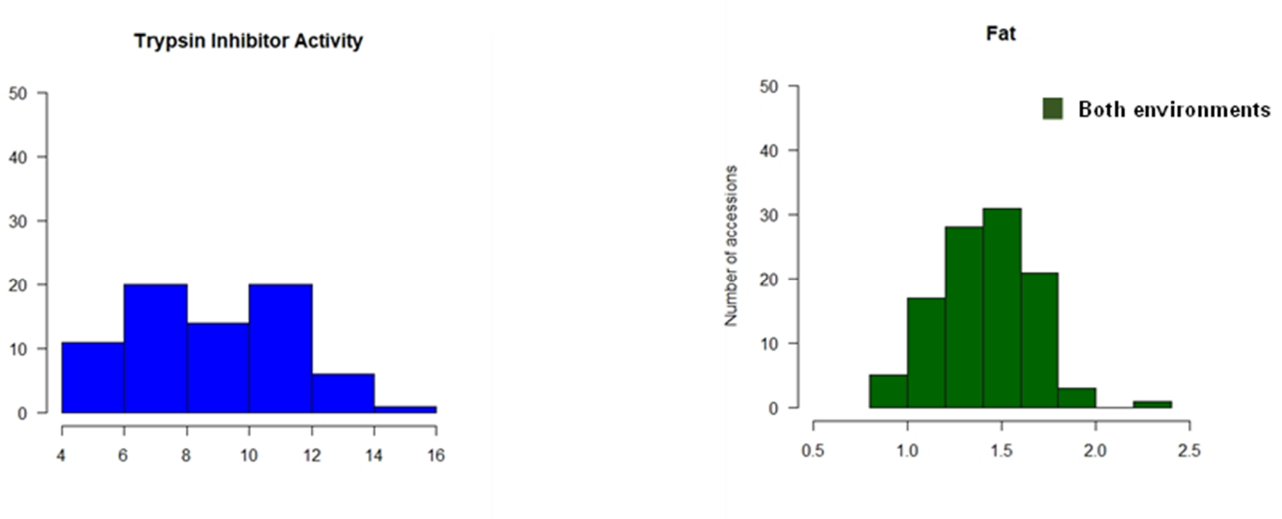

Figure S1. Histograms of the 24 nutritional composition and protein quality traits measured in a collection of 106 Portuguese common bean accessions. Traits measured in Cabrela and Córdoba environments are represented in different colors (grey—Cabrela, blue—Córdoba, green—both environments).

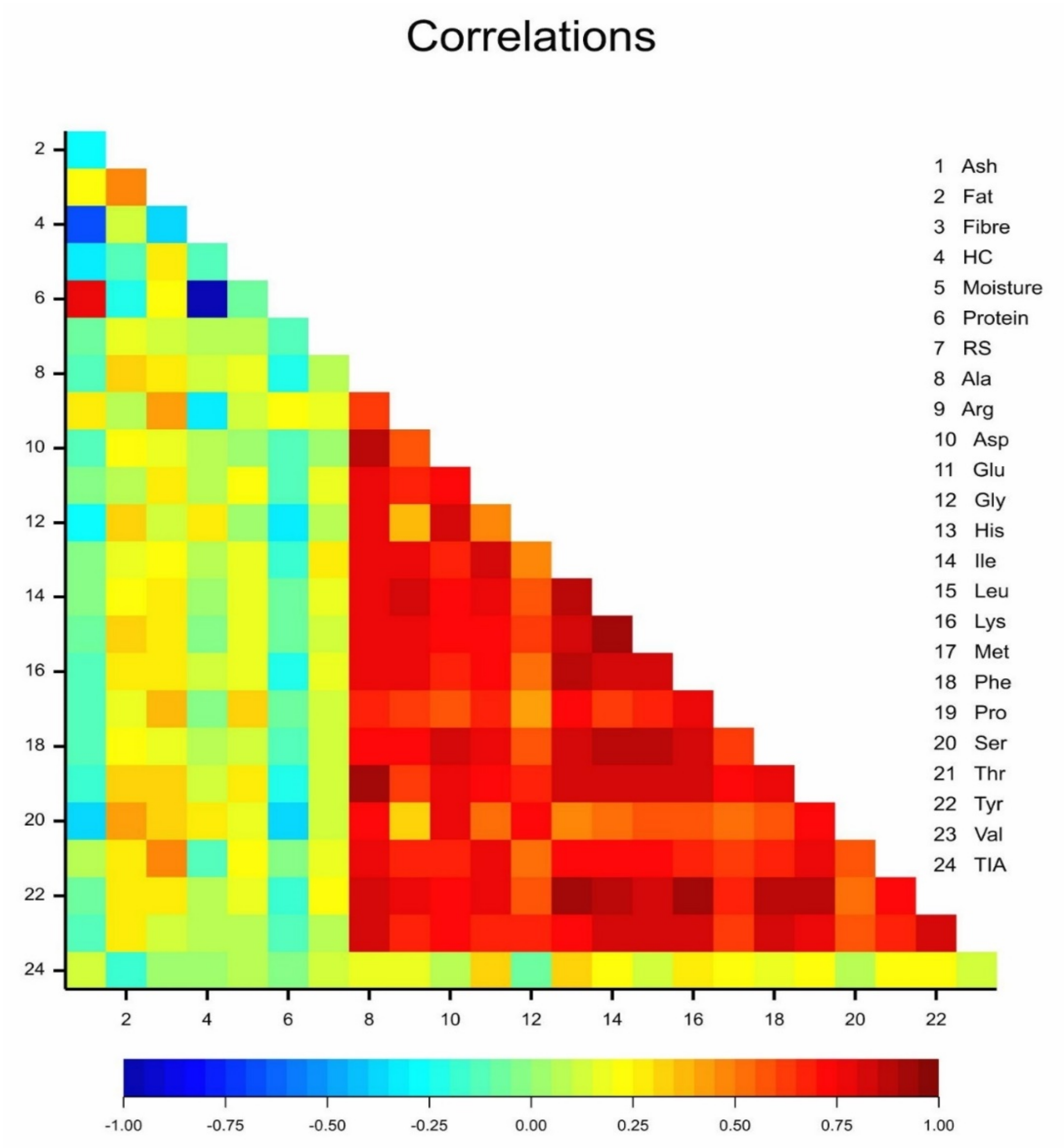

Figure S2. Pearson's correlations between the 24 nutritional composition and protein quality traits measured in the seeds of a collection of 72 Portuguese common bean accessions grown in the Córdoba environment. CH—carbohydrates; RS—resistant starch; Ala—alanine; Arg—arginine; Asp—aspartic acid; Glu—glutamic acid; Gly—glycine; His—histidine; Ile—isoleucine; Leu—leucine; Lys—lysine; Met—methionine; Phe—phenylalanine; Pro—proline; Ser—serine; Thr—threonine; Tyr—tyrosine; Val—valine; TIA—trypsin inhibitor activity. The "-t" after the trait's name indicates that data was transformed following a Box-Cox transformation.

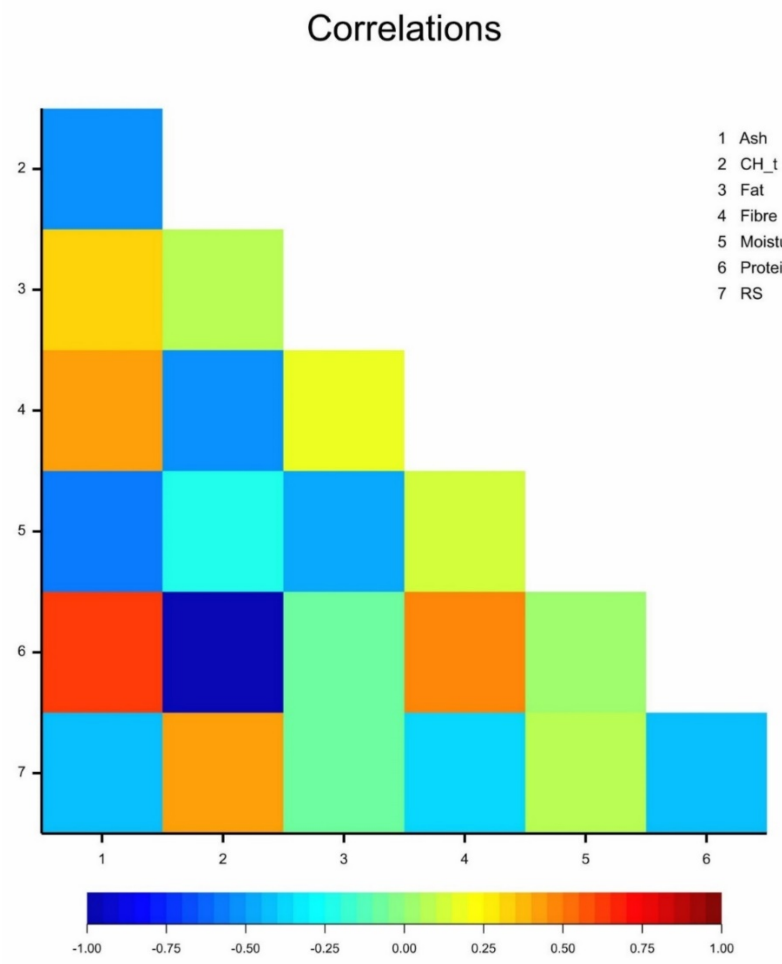

Figure S3. Pearson's correlations between the seven nutritional composition traits measured in the seeds of a collection of 78 Portuguese common bean accessions grown in the Cabrela environment. CH—carbohydrates; RS—resistant starch. The "_t" after the trait's name indicates that data was transformed following a Box-Cox transformation.

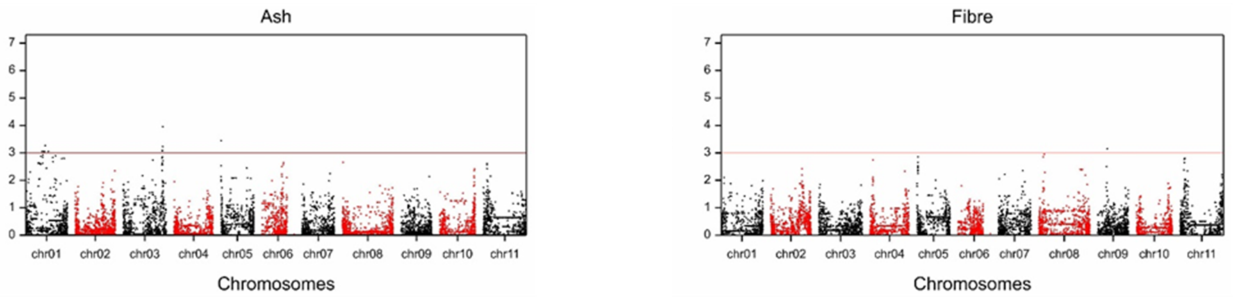

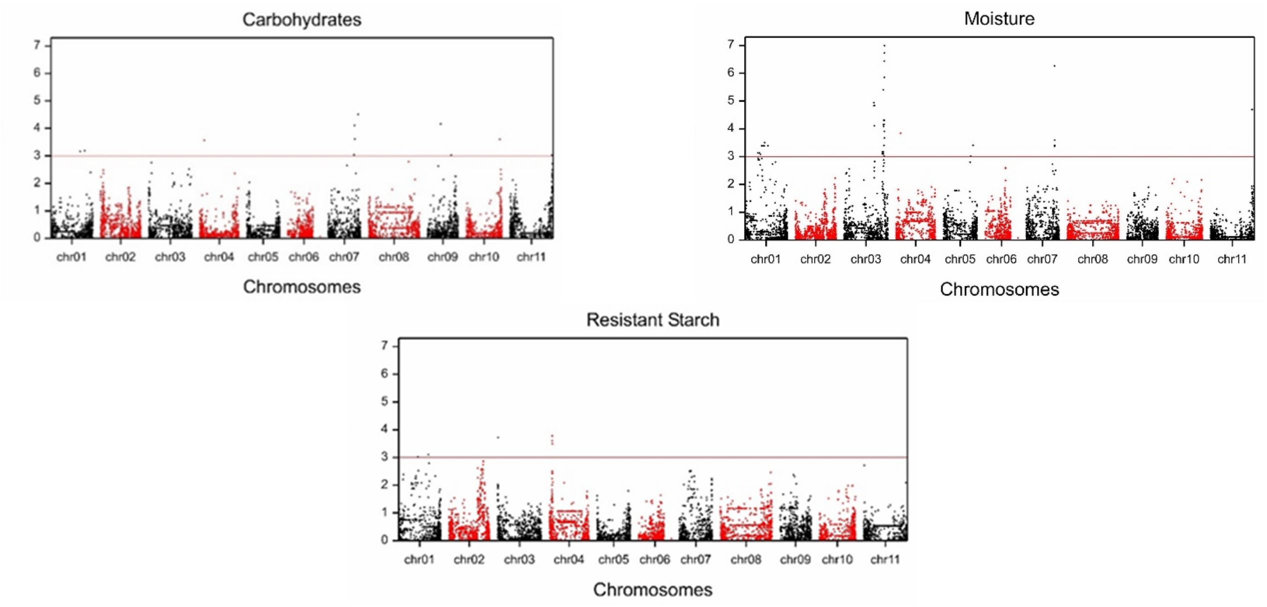

Figure S4. Manhattan plot depicting the genome-wide association results for ash, fiber, carbohydrates, moisture, and resistant starch content in common bean using 78 Portuguese accessions grown in the Cabrela environment. The y-axis represents the $-\log_{10}$ (p-value) of 9601 SNPs and the x-axis shows their chromosomal positions across the common bean genome. The horizontal red line indicates the significance threshold (p-value = 10^{-3}). The "-t" after the trait's name indicates that data was transformed following a Box-Cox transformation.

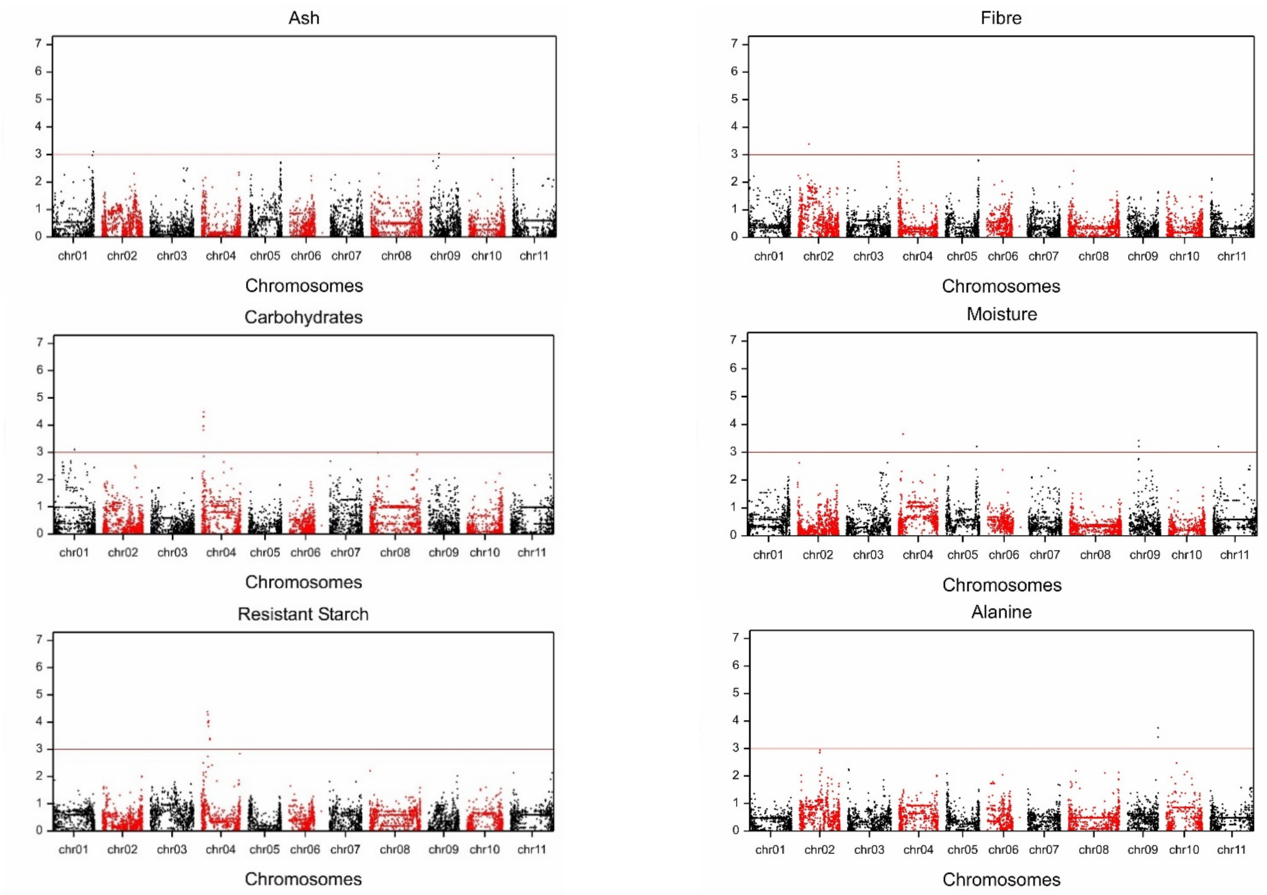

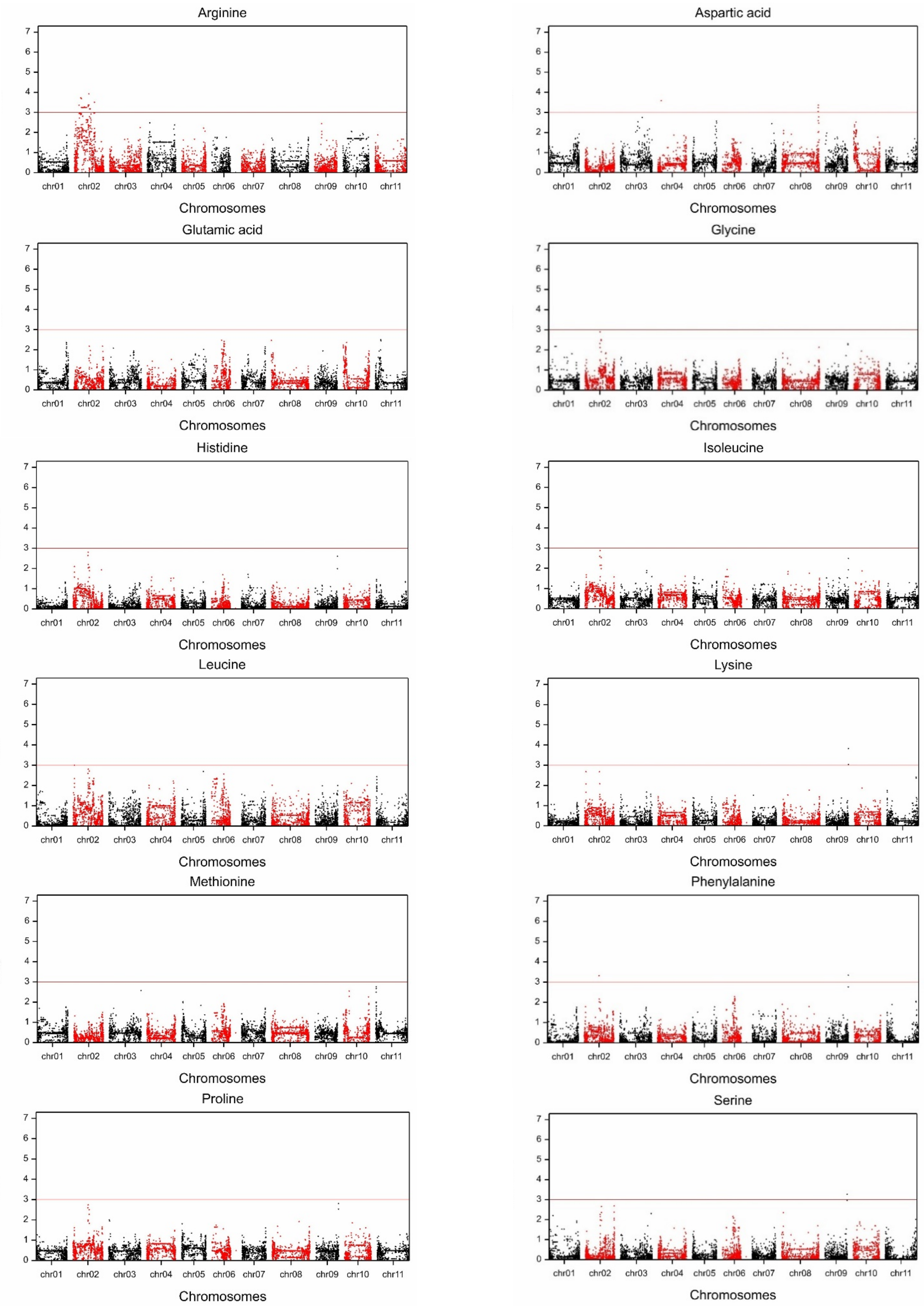
Arginine
Aspartic acid
Glutamic acid
Glycine
Histidine
Isoleucine
Leucine
Lysine
Methionine
Phenylalanine
Proline
Serine
0 1 2 3 4 5 6 7
chr01 chr02 chr03 chr04 chr05 chr06 chr07 chr08 chr09 chr10 chr11
Chromosomes

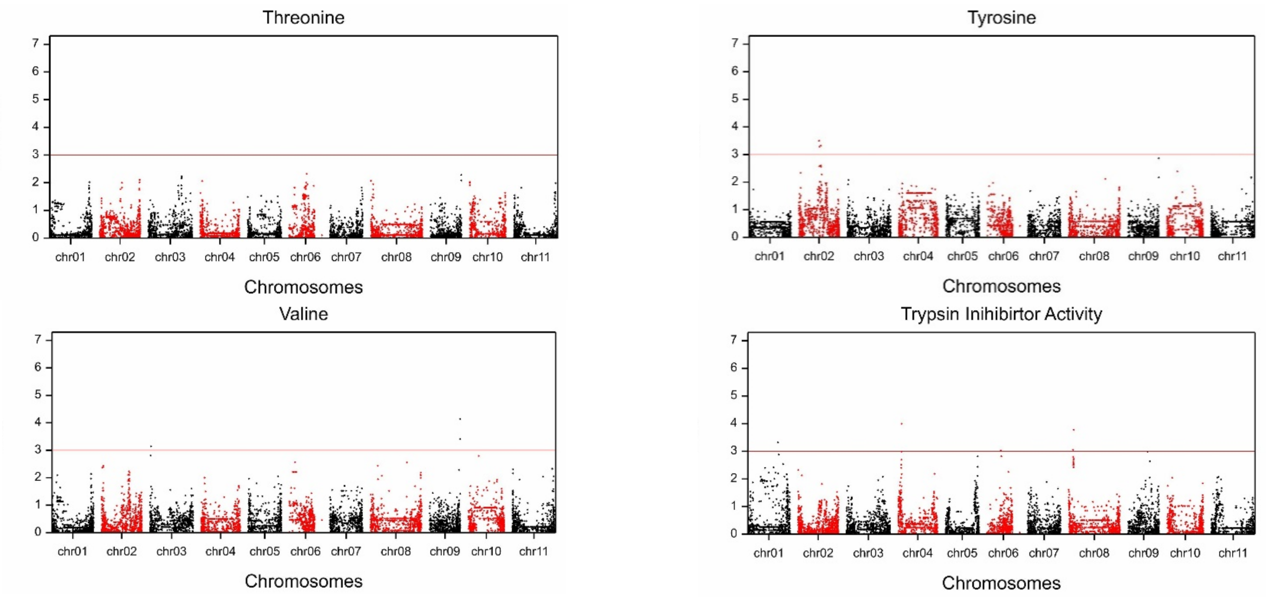

Figure S5. Manhattan plot depicting the genome-wide association results for ash, fiber, carbohydrates, moisture, protein, resistant starch, Alanine, Arginine, Aspartic acid, Glutamic acid, Glycine, Histidine, Isoleucine, Leucine, Lysine, Methionine, Phenylalanine, Proline, Serine, Threonine, Tyrosine, Valine and Trypsin inhibitor activity content in common bean using 94 Portuguese accessions grown in the Córdoba environment. The y-axis represents the $-\log_{10}$ (p-value) of 9601 SNPs and the x-axis shows their chromosomal positions across the common bean genome. The horizontal red line indicates the significance threshold (p-value = 10^{-3}). The "-t" after the trait's name indicates that data was transformed following a Box-Cox transformation.

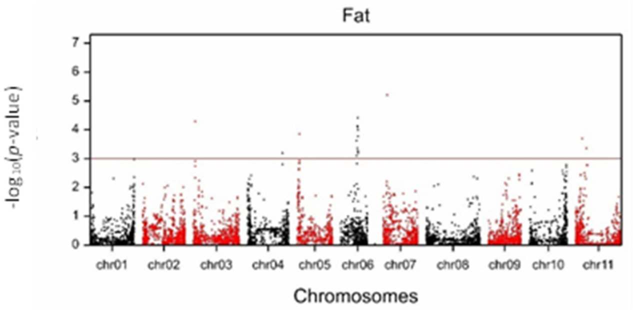

Figure S6. Manhattan plot depicting the genome-wide association results for fat content in common bean using 106 Portuguese accessions grown in the Cabrela and Córdoba environments. The y-axis represents the $-\log_{10}$ (p-value) of 9601 SNPs, and the x-axis shows their chromosomal positions across the common bean genome. The horizontal red line indicates the significance threshold (p-value = 10^{-3}). The "-t" after the trait's name indicates that data was transformed following a Box-Cox transformation.

Traits measured in Cabrela environment

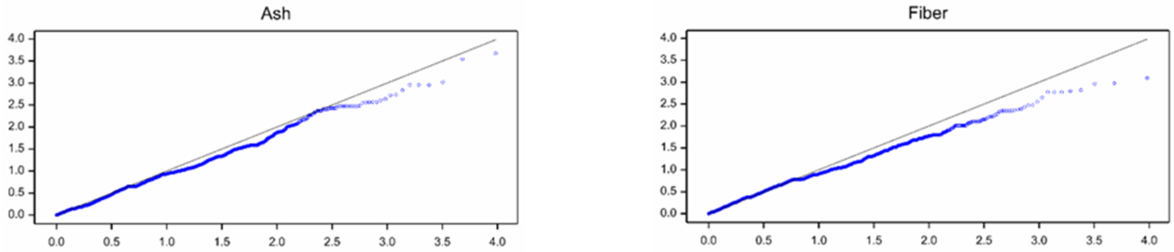

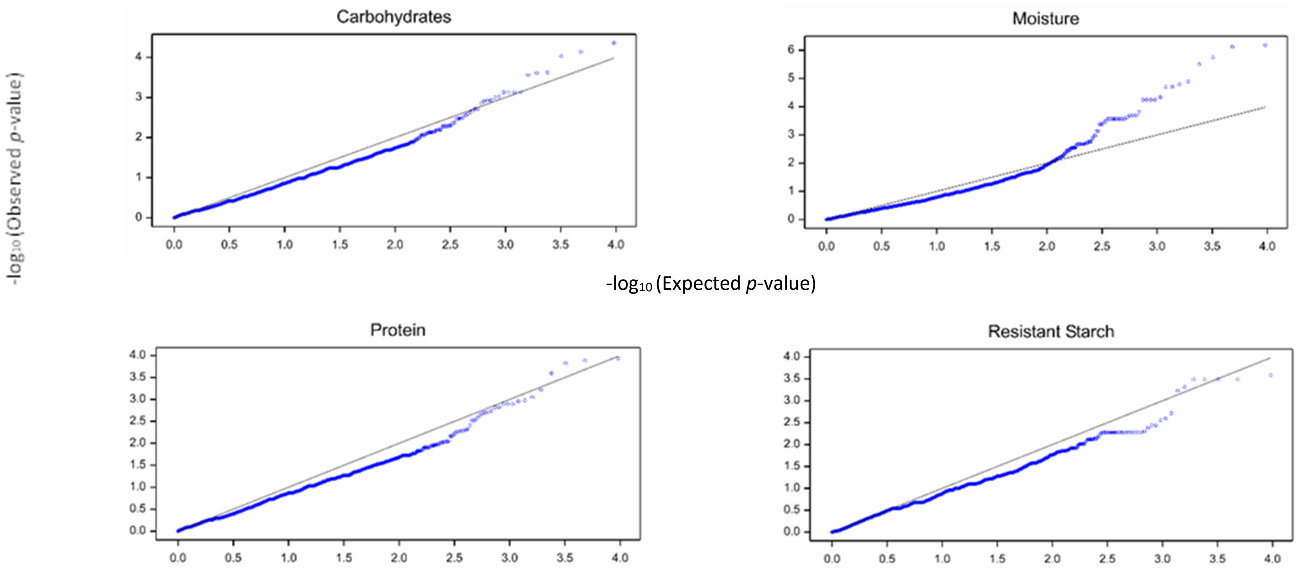

Traits measured in Córdoba environment

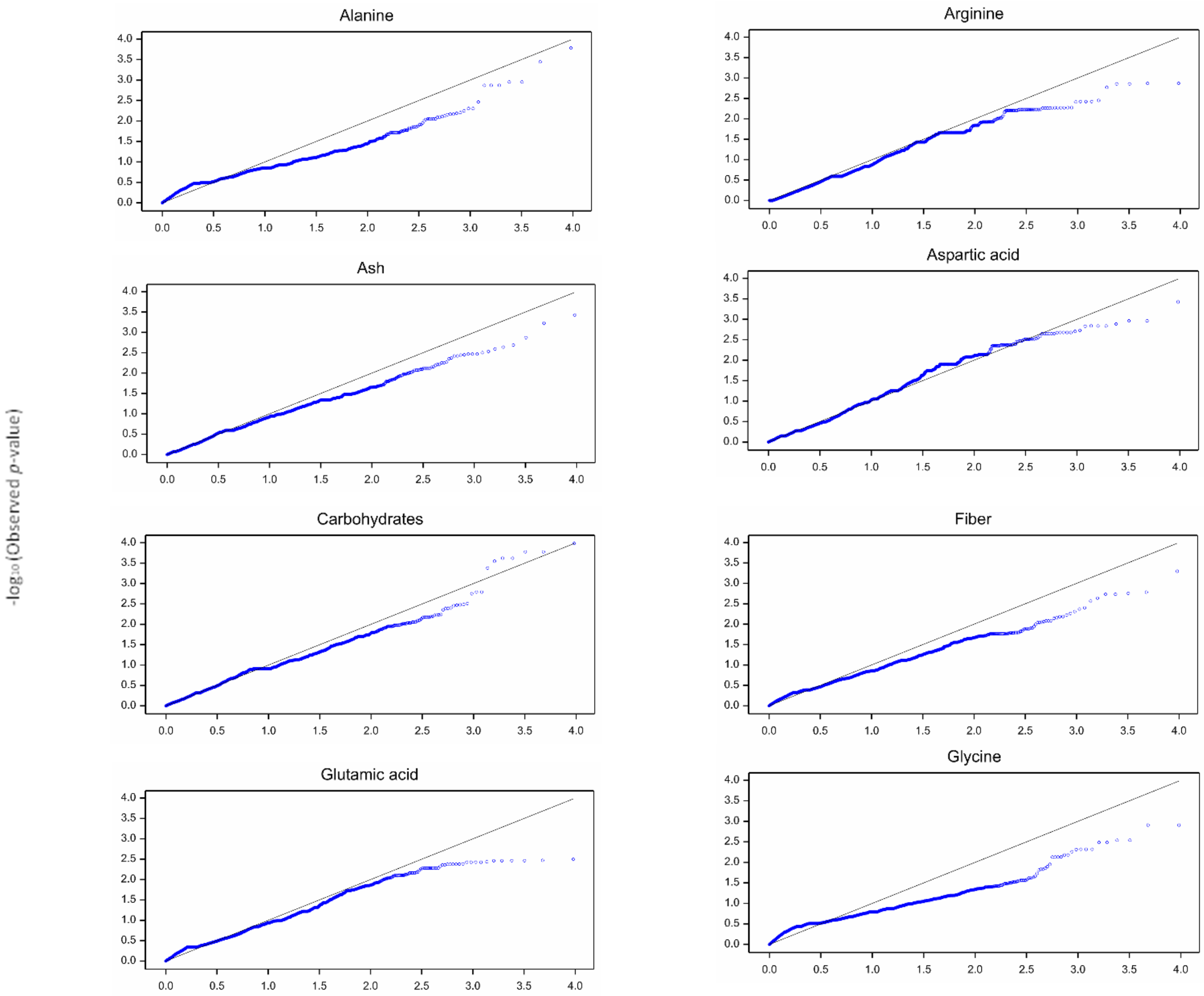

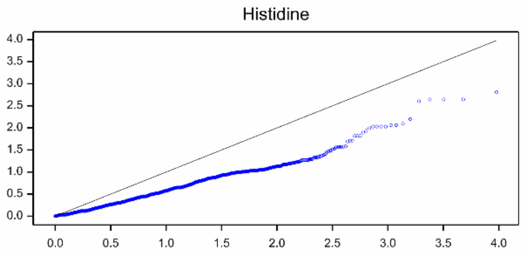
Histidine
0.0 0.5 1.0 1.5 2.0 2.5 3.0 3.5 4.0

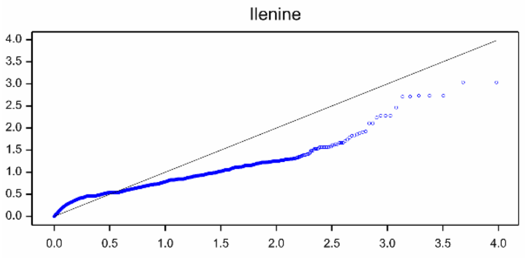
Ilenine
0.0 0.5 1.0 1.5 2.0 2.5 3.0 3.5 4.0

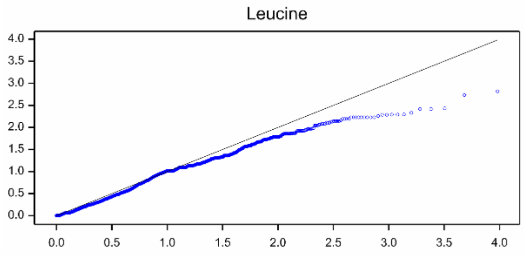
Leucine
0.0 0.5 1.0 1.5 2.0 2.5 3.0 3.5 4.0

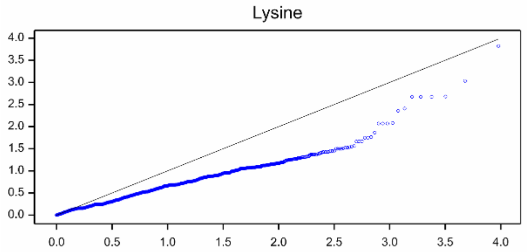
Lysine
0.0 0.5 1.0 1.5 2.0 2.5 3.0 3.5 4.0

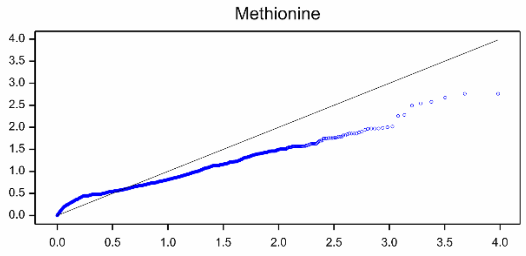
Methionine
0.0 0.5 1.0 1.5 2.0 2.5 3.0 3.5 4.0

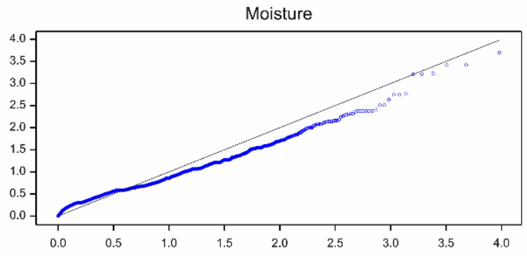
Moisture
0.0 0.5 1.0 1.5 2.0 2.5 3.0 3.5 4.0

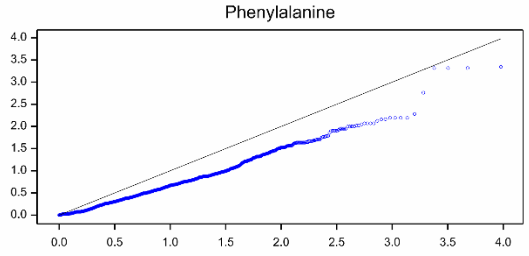
Phenylalanine
0.0 0.5 1.0 1.5 2.0 2.5 3.0 3.5 4.0

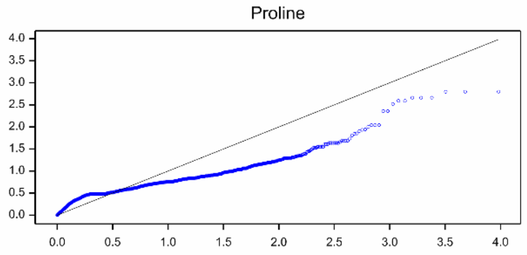
Proline
0.0 0.5 1.0 1.5 2.0 2.5 3.0 3.5 4.0

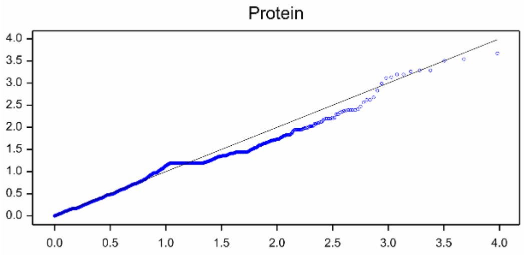
Protein
0.0 0.5 1.0 1.5 2.0 2.5 3.0 3.5 4.0

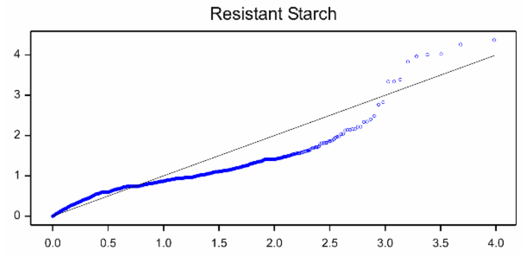
Resistant Starch
0 1 2 3 4
0.0 0.5 1.0 1.5 2.0 2.5 3.0 3.5 4.0

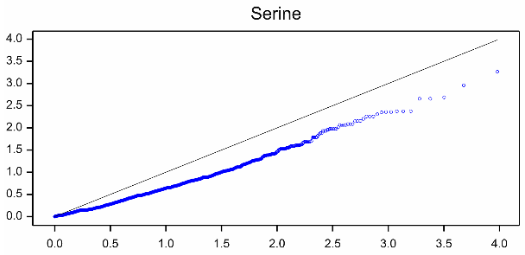
Serine
0.0 0.5 1.0 1.5 2.0 2.5 3.0 3.5 4.0

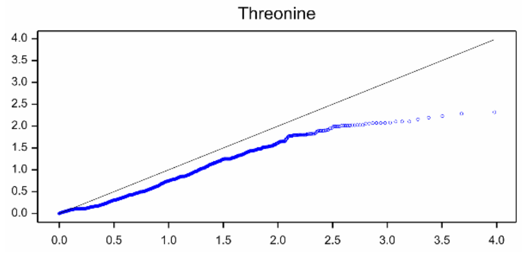
Threonine
0.0 0.5 1.0 1.5 2.0 2.5 3.0 3.5 4.0

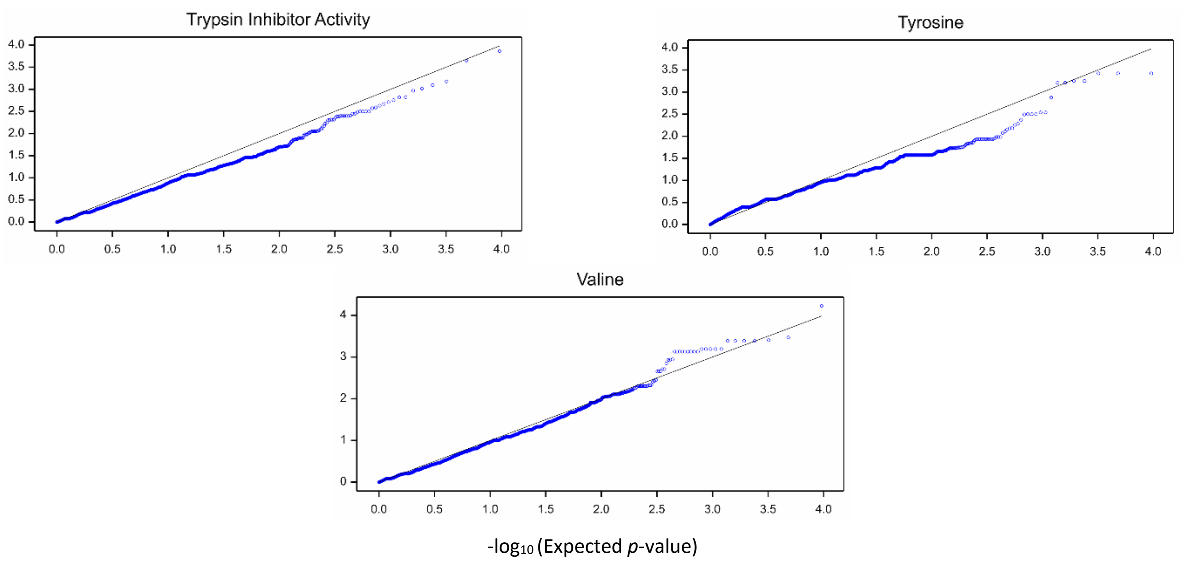

Figure S7. Quantile-quantile (Q-Q) plots for the SNP-trait associations of the 24 nutritional composition and protein quality-related traits measured in Cabrela, Córdoba, or both environments.
